# Effectiveness and safety of shortened intensive treatment for children with tuberculous meningitis (SURE): a protocol for a phase 3 randomised controlled trial evaluating 6 months of antituberculosis therapy and 8 weeks of aspirin in Asian and African children with tuberculous meningitis

**DOI:** 10.1136/bmjopen-2024-088543

**Published:** 2025-04-02

**Authors:** Julie Huynh, Chishala Chabala, Suvasini Sharma, Louise Choo, Varinder Singh, Naveen Sankhyan, Hilda Mujuru, Nhung Nguyen, Tung Huu Trinh, Phuc Huu Phan, Nguyen Viet Nhung, Kafula Lisa Nkole, Titiksha Sirari, Constantine Mutata, Elena Frangou, Anna Griffiths, Eric Wobudeya, Caitlin Muller, Sierra Santana, Evelyne Kestelyn, Lam Van Nguyen, Thanh Nguyen, Dai Tran, James A Seddon, Anna Turkova, Susan Abarca-Salazar, Robin Basu-Roy, Guy E Thwaites, Angela Crook, Suzanne T Anderson, Diana M Gibb, SURE trial team, Ennie Chidziva

**Affiliations:** 1 Oxford University Clinical Research Unit, Ho Chi Minh City, Ho Chi Minh, Vietnam; 2 Centre for Tropical Medicine and Global Health, Nuffield Department of Medicine, Oxford University, Oxford OX1 4BH, UK; 3 Department of Paediatrics, University of Zambia, Lusaka, Zambia; 4 University Teaching Hospitals-Children's Hospital, Lusaka, Zambia; 5 Lady Hardinge Medical College and Associated Kalawati Saran Children's Hospital, New Delhi, India; 6 MRC Clinical Trials Unit at UCL, London, UK; 7 Department of Pediatrics, Postgraduate Institute of Medical Education and Research, Chandigarh, India; 8 University of Zimbabwe Clinical Research Centre, Harare, Zimbabwe; 9 Department of Paediatrics, Pham Ngoc Thach Hospital, Ho Chi Minh City, Vietnam; 10 Children’s Hospital 2, Ho Chi Minh City, Vietnam; 11 Paediatric Intensive Care, Vietnam National Children’s Hospital, Hanoi, Vietnam; 12 The National Lung Hospital, Hanoi, Vietnam; 13 Makerere University—John Hopkins University Research Collaboration, Kampala, Uganda; 14 Center for Tropical Diseases, Vietnam National Children's Hospital, Hanoi, Vietnam; 15 Department of Infectious Disease, Imperial College London, London, UK; 16 Desmond Tutu TB Centre, Department of Paediatrics and Child Health, Stellenbosch University, Capetown, South Africa; 17 Clinical Research Department, London School of Hygiene & Tropical Medicine, London, UK; 18 Blizard Institute, Queen Mary University of London, London, UK

**Keywords:** Tuberculosis, Infectious diseases & infestations, Infectious disease/HIV, Paediatric infectious disease & immunisation, Randomized Controlled Trial, Clinical trials

## Abstract

**Introduction:**

Childhood tuberculous meningitis (TBM) is a devastating disease. The long-standing WHO recommendation for treatment is 2 months of intensive phase with isoniazid (H), rifampicin (R), pyrazinamide (Z) and ethambutol (E), followed by 10 months of isoniazid and rifampicin. In 2022, WHO released a conditional recommendation that 6 months of intensified antituberculosis therapy (ATT) could be used as an alternative for drug-susceptible TBM. However, this has never been evaluated in a randomised clinical trial. Trials evaluating ATT shortening regimens using high-dose rifampicin and drugs with better central nervous system penetration alongside adjuvant anti-inflammatory therapy are needed to improve outcomes.

**Methods and analysis:**

The Shortened Intensive Therapy for Children with Tuberculous Meningitis (SURE) trial is a phase 3, randomised, partially blinded, factorial trial being conducted in Asia (India and Vietnam) and Africa (Uganda, Zambia and Zimbabwe). It is coordinated by the Medical Research Council Clinical Trial Unit at University College London (MRCCTU at UCL). 400 children (aged 29 days to <18 years) with clinically diagnosed TBM will be randomised, using a factorial design, to either a 24-week intensified regimen (isoniazid (20 mg/kg), rifampicin (30 mg/kg), pyrazinamide (40 mg/kg) and levofloxacin (20 mg/kg)) or the standard 48-week ATT regimen and 8 weeks of high-dose aspirin or placebo. The primary outcome for the first randomisation is all-cause mortality, and for the second randomisation is the paediatric modified Rankin Scale (mRS), both at 48 weeks. Nested substudies include pharmacokinetics, pharmacogenetics, pathophysiology, diagnostics and prognostic biomarkers, in-depth neurodevelopmental outcomes, MRI and health economics.

**Ethics and dissemination:**

Local ethics committees at all participating study sites and respective regulators approved the SURE protocol. Ethics approval was also obtained from UCL, UK (14935/001). Informed consent from parents/carers and assent from age-appropriate children are required for all participants. Results will be published in international peer-reviewed journals, and appropriate media will be used to summarise results for patients and their families and policymakers.

**Trial registration:**

ISRCTN40829906 (registered 13 November 2018).

**STRENGTHS AND LIMITATIONS OF THIS STUDY**

- A randomised tuberculosis (TB) treatment-shortening trial in tuberculous meningitis (TBM) using a factorial design with an intensified anti-TB therapy and anti-inflammatory arm to answer two questions simultaneously.
- Conducted in Africa (3 countries and 3 centres) and Asia (2 countries and 6 centres) in high TB burden settings with clinical experience in diagnosing and managing TBM.
- Both mortality and neurodevelopmental (modified Rankin Scale and Liverpool Outcome Score) outcomes will be measured as primary and secondary outcomes, which will provide valuable insights into the efficacy of the shortened intensified therapy.
- This study includes nested substudies to examine novel diagnostic and prognostic biomarkers, long-term neurological sequelae, socioeconomic impact, compartmental pharmacokinetic data and cerebrospinal fluid pathophysiology studies.
- In this pragmatic trial, enrolled children have a clinical diagnosis of TBM, and although all have lumbar punctures, microbiological evidence of *Mycobacterium tuberculosis* is not mandated; this could result in some children without TBM being included.

## Introduction

### The burden of tuberculous meningitis (TBM)

Worldwide in 2023, an estimated 10.8 million people fell ill with TB, and 1.3 million died from it.^1^ While children made up 12% of those who developed tuberculosis (TB), they disproportionately accounted for 15% of TB deaths.^1^ Of these deaths, 14% occurred in children and adolescents living with HIV, while 76% were in children under 5 years of age.^2^ There are no estimates of the global burden of TBM owing to a lack of specific national surveillance data. Childhood TBM is often diagnosed late and is associated with high mortality and morbidity; 20% of children die, and 50% of survivors suffer long-term neuro sequelae despite anti-TB therapy (ATT).^3^ In a hospital setting, up to 15% of culture-confirmed childhood TB presents as TBM,^4^ and with decreasing cases of bacterial meningitis from other causes due to successful childhood vaccination programmes, TBM has become a more common cause of bacterial meningitis.^5^ Even in survivors without severe physical disability, cognitive and behavioural impairments are common, and the socioeconomic costs for families and healthcare systems are substantial.^6 7^ The unmet needs of children and adolescents affected by TBM have been recently highlighted in ‘Defeating Paediatric Tuberculous Meningitis: Applying the WHO Defeating Meningitis by 2030: Global Roadmap’.^8^ The WHO-recommended 12 month ATT regimen (2 months of isoniazid (H), rifampicin (R), pyrazinamide (Z) and ethambutol (E), followed by 10 months of isoniazid and rifampicin) was based on drugs and doses designed for pulmonary TB,^9^ and there is low-quality evidence to inform current guidelines. The 2010 WHO recommendation was to increase the doses of important backbone drugs, isoniazid (10–15 mg/kg) and rifampicin (10–20 mg/kg), for all forms of paediatric TB.^10^ However, limited data have been generated on the effect of these higher doses on childhood TBM,^11 12^ and doses remain inadequate to achieve appropriate cerebrospinal fluid (CSF) drug concentrations.^13^

Years of experience from a TB-endemic setting with a ‘Cape Town’ childhood TBM regimen comprising isoniazid (H, 20 mg/kg), rifampicin (R, 20 mg/kg), pyrazinamide (Z, 40 mg/kg) and ethionamide (Eto, 20 mg/kg) for 6 months reported low mortality.^14 15^ A recent systematic review and meta-analysis (1119 patients in 7 observational studies) evaluating treatment outcomes in children with TBM reported lower mortality in children receiving intensive 6 month versus standard 12 month regimens (5.5% vs 23.9%).^16^ This review was commissioned by WHO to inform the 2022 guidelines on the management of TB in children and adolescents and included a conditional recommendation that children and adolescents with drug-susceptible TBM could be treated with an intensive 6 month ‘Cape Town’ regimen as an alternative to the standard regimen while recognising that the former has not been subjected to a randomised controlled trial. The existing strong recommendation for the 12 month regimen remains.^17 18^ WHO and childhood TB experts globally have been calling for trials in TBM to evaluate shorter, intensive regimen alternatives; authors of a recent Cochrane systematic review repeated this call for both adults and children.^19^,^21^

### High-dose rifampicin

Rifampicin is critical in the early days of treatment, and high doses (eg, 35 mg/kg) are well tolerated.^22^,^24^ Pharmacokinetic (PK)-pharmacodynamic (PD) analyses show that higher exposures of rifampicin are essential to decrease time to culture conversion.^25^

Children have lower plasma exposure to rifampicin compared to adults with the same mg/kg dosing. Recent PK data from 100 Vietnamese children with TBM receiving a 10 mg/kg rifampicin dose found few with a target therapeutic plasma level, and nearly all had CSF concentrations well below the wild-type minimal inhibitory concentration (MIC).^26^ A small randomised trial in India and Malawi (TBM-KIDS, Tuberculous Meningitis in Kids study) used 30 mg/kg oral rifampicin daily, based on PK-PD modelling, showing that this dose was required to achieve optimal CSF rifampicin exposures in children.^27^ Further, in a paediatric PK and safety study (Opti-Rif, Optimizing Rifampicin Trial), 2 weeks of higher doses of rifampicin (up to 60–75 mg/kg) were safe and achieved target serum exposures.^28^ Although it is well known that children require higher mg/kg doses than adults to achieve target exposures, the safety of high rifampicin doses over longer durations is not yet established. Phase 3 trials evaluating the potential mortality benefit of high-dose rifampicin (30–35 mg/kg) are ongoing in adults and none in children (table 1).

**Table 1:** Ongoing adult trials evaluating anti-TB drug regimens and adjunctive therapies in TBM

Trial and registry number	Population	Trial design	Study intervention	Setting	Sample size	Trial status
Anti-TB chemotherapy						
HARVEST ISRCTN15668391	Aged ≥18 yearsHIV co-infected and uninfected	Phase 3Randomised, parallelDouble-blindPlacebo-controlled	High-dose rifampicin (35 mg/kg) for 8 weeks	Indonesia, South Africa and Uganda	500 (planned)	Recruiting. Estimated completion—June 2025
ALTERNCT04021121	Aged >18 yearsHIV co-infected and uninfected	Phase 2Randomised, factorialOpen label	High-dose rifampicin (35 mg/kg) ANDLinezolid (1200 mg)for 4 weeks	Uganda	40	Recruitment completed. Results awaited
SIMPLENCT03537495	Aged ≥18 yearsHIV co-infected and uninfected	Phase 2Randomised, parallelOpen label	High-dose rifampicin (35 mg/kg) ANDLinezolid (600 mg or 1200 mg)for 14 days	Indonesia	36	Recruitment completed. Results awaited
IMAGINE-TBMNCT05383742	Aged ≥15 yearsHIV co-infected and uninfected	Phase 2Randomised, parallelOpen label	High-dose rifampicin (35 mg/kg) ANDHigh-dose isoniazid (15 mg/kg) ANDLinezolid (1200 mg) for 2 weeks, followed byHigh-dose isoniazid (10 mg/kg) along with other study drug interventions at the same doses for a further 6 weeks, THENHigh-dose rifampicin (35 mg/kg) ANDHigh-dose isoniazid (10 mg/kg) only for 16 weeks to complete a 24-week study treatment	Brazil, India, Kenya, Malawi, Mexico, Peru, Philippines, South Africa, Tanzania, Thailand, Vietnam and Zimbabwe	330 (planned)	Recruiting. Estimated completion—May 2027
Optimising ATT in adults with TBM based on NAT-2 genotypingNCT03787940	Aged 18–65 years	Phase—n/aRandomised, parallelDouble-blind	Dosing of isoniazid stratified by NAT-2 statusHigh-dose isoniazid 900 mg for 3 months for rapid acetylators	China	676 (planned)	Unknown status
Adjunctive therapy						
LAST-ACTNCT03100786	Aged ≥18 yearsHIV uninfected only	Phase 3Randomised, parallelDouble-blindPlacebo-controlled	Adjunctive dexamethasone (intravenous, followed by oral, according to disease severity at start of treatment) for 6–8 weeks	Vietnam	720	Recruitment completed. Results awaited.
A study for evaluation of the use of indomethacin in patients with TBMCTRI/2018/02/011722	Any ageHIV uninfected only	RandomisedOpen label	Indomethacin, in addition to the standard of care	India	300 (planned)	Recruiting. Estimated completion—unknown
TIMPANINCT05590455	Aged ≥18 yearsHIV co-infected only	Phase 2Randomised, parallelOpen label	Adalimumab 40 mg sc every 2 weeks for 10 weeks, in addition to the standard of care	Brazil, Mozambique and Zambia	130 (planned)	Recruiting. Estimated completion—December 2025
Anti-TB chemotherapy and adjunctive therapy						
INTENSE TBMNCT04145258	Aged ≥15 yearsHIV co-infected and uninfected	Phase 3Randomised, factorialPartially blindedPlacebo-controlled (aspirin treatment)	High-dose rifampicin (35 mg/kg)Linezolid (1200 mg and then 600 mg)ANDAdjunctive aspirin (200 mg) for 8 weeks	Ivory Coast, Madagascar, Uganda and South Africa	768 (planned)	Recruiting. Estimated completion—April 2026

ATTantituberculosis therapyNAT-2N-acetyltransferase type 2 scsubcutaneousTBtuberculosisTBMtuberculous meningitis

### High-dose isoniazid

Isoniazid has excellent CSF penetration and high early bactericidal activity (EBA), killing 95% of mycobacteria within the first few days.^29^ The ‘Cape Town’ regimen consists of isoniazid at a dose of ~20 mg/kg, which usually exceeds the MIC of susceptible strains and can overcome low-level isoniazid resistance.^30^ Nested PK-PD studies in an adult TBM treatment trial intensified with rifampicin (15 mg/kg) and levofloxacin (20 mg/kg) found that low isoniazid exposure predicted death and was linked to a fast acetylator phenotype.^31^ High doses of isoniazid to improve outcomes in TBM are currently being investigated in adult TBM trials (table 1).

### Levofloxacin as an alternative fourth agent

Levofloxacin (L) penetrates the CSF well, with concentrations reaching up to 70% of plasma concentrations.^32^ Unlike ethionamide (used in the ‘Cape Town’ regimen, which is conditionally recommended by WHO^18^), levofloxacin retains activity against mycobacteria with all types of isoniazid resistance, including *inhA* promoter mutations against which ethionamide is ineffective. Additionally, levofloxacin has an EBA comparable to isoniazid,^33^ which is better tolerated (significant nausea and vomiting are associated with ethionamide), easier to access, cheaper and available as a dispersible paediatric tablet.^34^ While tendon rupture, arthropathy and cardiac safety signals associated with levofloxacin are a concern in adults, these adverse events (AEs) have rarely been reported in children.^35^ Its use in large childhood TB trials, including TBM-KIDS and TB-CHAMP (tuberculosis child multidrug-resistant preventive therapy trial), has shown it to be very safe with few severe adverse effects.^22 36^

### High-dose aspirin as an anti-inflammatory agent

Arterial ischaemic cerebral stroke caused by hyperinflammation and hypercoagulability is a key cause of irreversible neurological damage in TBM and may not be prevented by adjunctive corticosteroids.^37^,^39^ Aspirin may have a role in treating the host inflammatory response in TBM by inhibiting cyclo-oxygenase, reducing thromboxane and prostaglandin production and triggering the production of pro-resolving mediators that actively promote the resolution of inflammation.^40^

Two small trials in adults have evaluated aspirin with promising results. The first (n=118) demonstrated the benefit of the risk of stroke (absolute risk reduction: 19.1%) and reduction in mortality of low-dose aspirin (150 mg) compared with placebo (21.7% vs 43.4%, p=0.02).^41^ The second was a phase 2, double-blind trial of placebo versus low-dose (81 mg/day) versus high-dose (1000 mg/day) adjunctive aspirin in 120 Vietnamese adults with TBM, which suggested a potential reduction in new infarcts and deaths in the aspirin-treated participants with microbiologically confirmed TBM, with 11/32 (34.4%) events in placebo versus 4/27 (14.8%) in aspirin 81 mg versus 3/28 (10.7%) in aspirin 1000 mg (p=0.06).^42^

The only paediatric trial (n=146) evaluating high-dose and low-dose aspirin against placebo found no impact on survival and no significant difference in clinical neurological outcomes. However, more children who received high-dose aspirin (100 mg/kg/day) had severe disease at baseline, and none developed new hemiplegia—a beneficial effect potentially due to the aspirin and one that requires further investigation.^43^

## Methods and analysis

### Study design

The Shortened Intensive Treatment for Children with Tuberculous Meningitis (SURE) trial is a phase 3, randomised, partially blinded, factorial trial of 6 months of intensified ATT and 8 weeks of anti-inflammatory treatment for HIV-positive and HIV-negative African and Asian children with TBM. Partially blinded refers to the double blinding of the anti-inflammatory (aspirin vs placebo) randomisation and the open-label ATT randomisation (6 months vs 12 months). It opened for recruitment in March 2021, and the last child will complete 72 weeks of follow-up by December 2025.

### Rationale for a paediatric TBM clinical trial

The SURE trial compares the 12-month WHO standard with a 6-month intensified ATT regimen, modified from the ‘Cape Town’ regimen comprising rifampicin (30 mg/kg), isoniazid (20 mg/kg), pyrazinamide (40 mg/kg) and levofloxacin (20 mg/kg). Halving the recommended treatment duration while optimising treatment doses may decrease risks of long-term drug toxicity, improve treatment adherence, protect against the development of drug-resistant strains and reduce the burden on families and TB control programmes. There is no evidence to date that 6-month regimens result in increased relapse rates. If an 8-week anti-inflammatory dose of aspirin reduces neurodisability, then this safe, cheap and widely available adjunctive agent could also improve TBM outcomes in children.

### Main study objectives

1. To determine whether the standard 12-month ATT regimen for treating paediatric TBM can be reduced, with similar efficacy and safety, to a 6-month intensified regimen of anti-TB drugs in children aged 29 days to less than 18 years.
2. To determine whether adjunctive aspirin for 8 weeks of treatment reduces TBM-related neurodisability with minimal or no toxicity.

### Primary outcomes

The primary outcome for randomisation 1 (ATT regimen comparison) is all-cause mortality at 48 weeks. The primary outcome for randomisation 2 (aspirin/placebo comparison) is the modified Rankin Scale (mRS), dichotomised as death (score 6 out of 6) or severe impairment (score 5) versus the rest (score 0–4) at 48 weeks.

### Secondary outcomes

Secondary efficacy and safety outcomes for both randomisations are detailed in table 2.

**Table 2:** Secondary outcomes

Neurodevelopmental outcome	mRS at 24, 48 (randomisation 1) and 72 weeks.
Mortality	All-cause mortality at 72 weeks.
TB treatment failure	Clinical or microbiological relapse of TBM and/or TB disease at other sites by 72 weeks.
Specific AEs	Any grade 3 or 4 (as per DAIDs) clinical (including arthralgia and arthritis due to levofloxacin use) or laboratory AE. AE (any grade) leading to treatment modification. Gastrointestinal bleeding (any grade) due to increased risk with high-dose aspirin. DILI of grade 2 or more. Development of obstructive hydrocephalus. Tendonitis or tendon pain (any grade).
Acquired drug resistance	Drug resistance identified on genotypic/phenotypic DST—any suspected relapse with confirmed or suspected drug resistance.
Adherence to treatment	Adequate TB treatment adherence is defined as having taken at least 80% of doses.
Acceptability of treatment	Acceptability (taste, pill burden and drug administration) at 8, 24 and 48 weeks by the 4 group randomisation strata.
Viral load in children with HIV	Suppressed HIV viral load <50 copies/mL and change in CD4 count from baseline to 24, 48 and 72 weeks in children living with HIV.

AEadverse eventCD4CD4 T lymphyocyteDAIDsDivision of AIDS grading of severityDILIdrug-induced liver injuryDSTdrug susceptibility testingmRSmodified Rankin ScaleTBtuberculosisTBMtuberculous meningitis

### Setting and population

SURE is being conducted in Asia (India and Vietnam) and Africa (Uganda, Zambia and Zimbabwe). Recruitment sites include Postgraduate Institute of Education and Medical Research, Chandigarh; Lady Hardinge Medical College, New Delhi; Pham Ngoc Thach Hospital, Ho Chi Minh City; Children’s Hospital 2, Ho Chi Minh City; Vietnam National Lung and TB Hospital, Ha Noi; Vietnam National Children’s Hospital, Ha Noi; University Teaching Hospital—Children’s Hospital, Lusaka; University of Zimbabwe Clinical Research Centre, Harare and Makerere University—John Hopkins University Research collaboration, Kampala, Uganda. Eligible participants with TBM are enrolled if they meet the study criteria summarised in Box 1, as per SURE protocol V3.0 (4th December 2023).

Box 1 **Inclusion and exclusion criteria**

**Inclusion criteria**

- Aged between 29 days and less than 18 years.
- Weight ≥3 kg.
- Symptoms compatible with TBM.
- CSF results are compatible with TBM, with or without AFB on microscopy or *Mycobacterium tuberculosis* on GeneXpert (or other validated NAATs), *or* the physician believes the child needs immediate initiation of anti-TB drugs.
- Known (or pending confirmation of) HIV status.
- The parent/legal guardian gives informed, written consent.
- Agree for a CSF sample to be collected and processed for chemistry, microscopy, Ziehl-Neelsen or auramine stain and mycobacterial culture (in process), and, where available, Xpert or Xpert Ultra prior to commencing treatment or when clinically stable to do so. Where patients have already been started on ATT, prescreening CSF should be available.
- The participant’s carer/parent can comply with the protocol requirements in the opinion of the site investigator.

**Exclusion criteria**

- Recent contact (last 12 months) with known or suspected rifampicin-resistant TB.
- Proven drug resistance to rifampicin in the child.
- On ATT for >21 days.
- Severely moribund; high risk of death within 24 hours based on clinical judgement.
- History or presence of known allergy or other contraindication to any first-line anti-TB drugs, corticosteroids or aspirin.
- Pregnancy.
- History of gastrointestinal bleeding or bleeding diathesis.
- Active clinical infection with influenza or varicella.
- Grade 4 liver toxicity (ALT and/or AST ≥10× upper limit of normal) or other contraindications* for taking part in the trial.

AFB, acid fast bacilli; ALT, alanine transaminase; AST, aspartate aminotransferase; ATT, antituberculosis therapy; CSF, cerebrospinal fluid; HIV, human immunodeficiency virus; NAAT, nucleic acid amplification test; TB, tuberculosis; TBM, tuberculous meningitis. *Other contraindications are rare and allow the attending physician to make a decision at their own discretion about whether a participant is eligible. For example, a history of long QT syndrome, being already on several high-risk drugs and concern for an increased risk if randomised to short ATT, which includes levofloxacin.

Following low recruitment rates in the early phase of the trial (in part due to the COVID-19 pandemic), eligibility criteria were expanded to increase participant recruitment in February 2022. The age range was broadened from 29 days to 15 years to later include children up to 18 years of age. The duration of ATT as an exclusion criterion was also expanded from ≤7 days to ≤21 days.

### Screening procedures

Parents/carers of children with presumptive TBM are invited to participate after the child is identified as potentially eligible. Informed consent is obtained from parents/guardians prior to trial participation, with assent additionally sought from children who are developmentally and cognitively capable, as per local ethics committee guidance. The consent information is provided in the local language using locally translated patient information sheets and consent forms (online supplemental material). Different treatments and possible toxicities by arm will be explained before performing trial-specific procedures or any blood is taken for the trial. If a child is too unwell (eg, comatose) to provide assent, parent/guardian consent is sufficient, and assent, if developmentally appropriate, is taken at a later date when the child’s condition improves. Participants undergo a screening assessment, including TB symptoms and risk factors, and a physical examination, including anthropometric evaluations, seizure frequency, Glasgow coma scale, focal neurology and TBM severity (Medical Research Council grade) (online supplemental table S1a). CSF is collected (unless already collected <7 days prior to screening) by lumbar puncture or ventricular shunt for microscopy, biochemistry and TB diagnostics, that is, acid fast stain, Xpert MTB/RIF (Mycobacterium tuberculosis/rifampicin), Xpert Ultra or Truenat (whichever is available) and mycobacterial culture. Safe CSF volumes are collected according to published guidance.^44^ Cultured isolates are sent for drug susceptibility testing (DST) of *Mycobacterium tuberculosis*, in addition to resistance (if any) already identified on nucleic acid amplification testing. A gastric aspirate or alternative respiratory sample are collected prior to commencing ATT or within 7 days of commencement.

TBM diagnostic certainty is determined retrospectively based on the established uniform case definition for TBM.^45^ For children living with HIV and CD4 counts of <100 cells/mL, India Ink or cryptococcal antigen testing is performed. Phenotypic DST is done on positive cultures in real time. Repeat TB diagnostics are completed at the time of suspected TB treatment failure or relapse/recurrence. Cultures positive for *M. tuberculosis* are stored for strain genotyping. Participants have a chest X-ray and neuroimaging of the brain at enrolment or as soon as clinically feasible.

### Randomisation and treatment allocation

**Figure 1:**
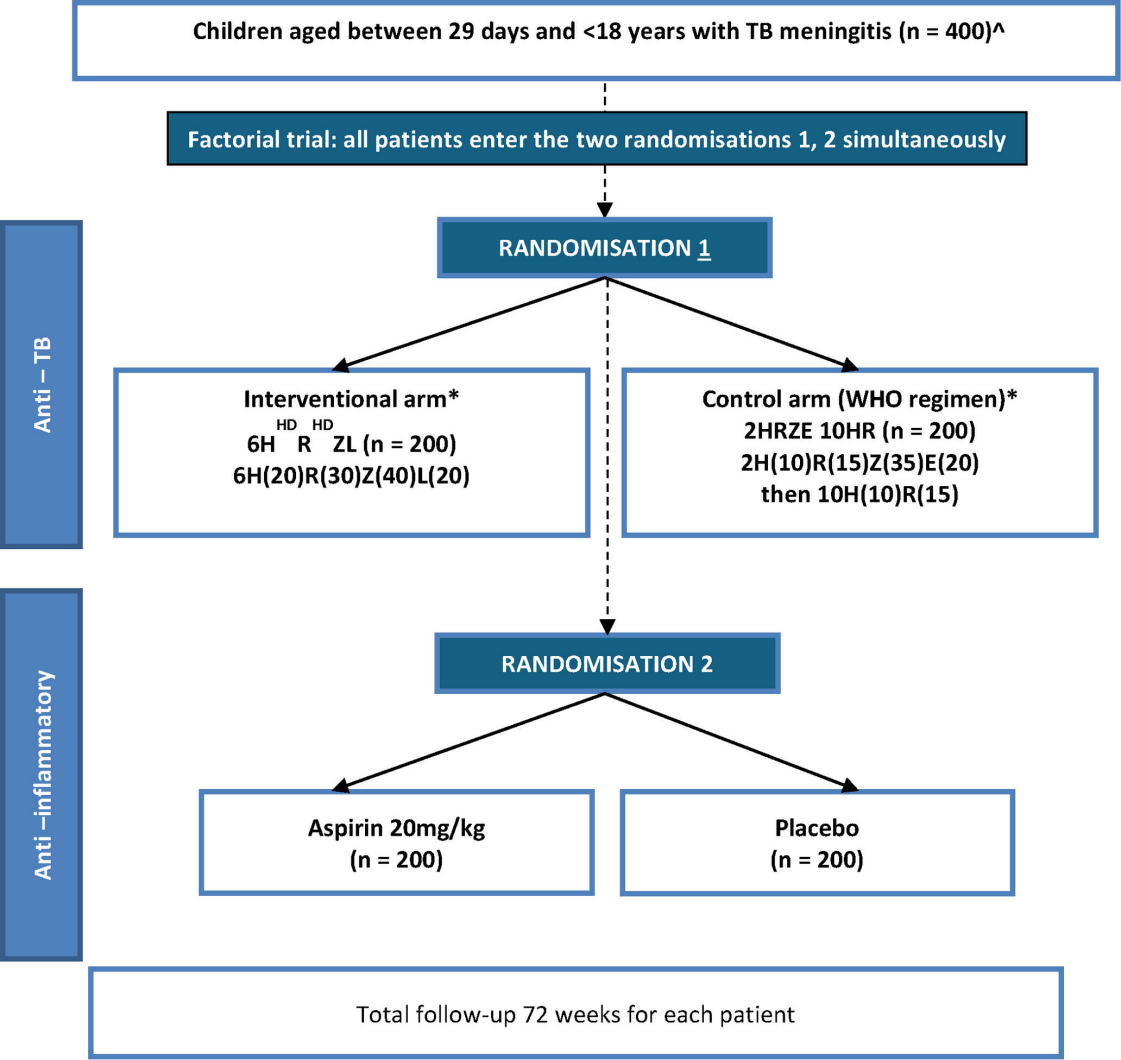
Trial entry, randomisation and treatment. HD, high-dose; E, ethambutol; H, isoniazid; L, levofloxacin; R, rifampicin; TB, tuberculosis and Z, pyrazinamide. ˆ Target of 400. If planned recruitment to 400 is not possible, the modified target of 300 would be acceptable to allow 80% power and 350 to allow 85% power. * reduced mg/kg dosing used for children 25 kg and over. Both randomisations use minimisation with a random element and 1:1 allocation ratio.

Randomisations occur immediately once eligibility criteria are confirmed and informed consent is obtained. Participants are randomised twice simultaneously (figure 1, *factorial design*) using a web-based, electronic randomisation system controlled through an authorised username and password to:

1. 6-month (24 weeks) intensive ATT regimen or a standard 12-month (48 weeks) ATT regimen in a 1:1 ratio (open-label).
2. 8 weeks of aspirin or matching placebo, in a 1:1 ratio (double-blinded).

Randomisation is a dynamic and concealed process; no preprepared lists are generated. Randomisation allocations are held securely within the Medical Research Council Clinical Trial Unit (MRCCTU) randomisation system and the Drug Supply Management System (DSMS). To randomise a participant, information is entered into an online trial database, and eligibility is automatically checked. On successful randomisation, the clinician prescribes allocated trial drugs. In all cases, neither the participant nor the investigator is notified of the treatment allocation of aspirin or placebo.

Aspirin and placebo are packed and prelabelled according to Good Manufacturing Practice guidelines with a code to maintain blinding for both trial site staff and participants recruited.

Since blinding of aspirin allocation is critical to the integrity of the study, all children are managed with the assumption that they receive aspirin. Unblinding during the trial is strongly discouraged unless it is a medical emergency and would alter clinical management. An emergency unblinding system for blinded randomisation can only be performed by a site principal investigator (PI) or allocated co-investigator through the DSMS. One unblinded delegated statistician has regular access to the treatment allocations for routine data querying and safety reporting purposes.

### Anti-TB regimen (randomisation 1)

All TB study drugs are fixed-dose combinations or paediatric dispersible formulations with the exception of levofloxacin (250 mg tabs) and pyrazinamide (400 mg tabs). Both the control and intervention arms use WHO-modified weight bands, which are adapted to achieve adequate doses and consistency across both treatment arms. In the intervention arm, children weighing >25 kg have a lower mg/kg target dosing compared with children <25 kg with isoniazid (10 mg/kg), rifampicin (20 mg/kg) and pyrazinamide (30 mg/kg) (online supplemental table S2a and S2b (*intervention*) and online supplemental table 3a–3d (*control*)). All trial ATT drugs for all sites are manufactured by a single source.

### Adjunctive aspirin (randomisation 2)

Aspirin at a target dose of 20 mg/kg once daily for 8 weeks is dosed according to WHO-modified weight bands using dispersible aspirin 80 mg tablets. Placebo is matched in appearance, taste and odour. The same WHO-modified weight bands as for randomisation 1 are used (online supplemental table S4).

### HIV co-infection

For children living with HIV, antiretroviral therapy (ART) is commenced within 4–8 weeks of ATT, unless already on treatment and in line with WHO guidance (the timing was adjusted from 2-8 weeks used at the start of the trial). ART is provided by country programmes, and drugs not recommended for simultaneous use with rifampicin are replaced with alternatives as per the WHO guidelines 2016. All children with TB and HIV co-infection receive cotrimoxazole prophylaxis for other infections and follow WHO/local guidelines on the use of any other prophylactic medications.

### Concomitant drug therapy

#### Adjunctive corticosteroids

All children receive either oral prednisolone at 2–4 mg/kg daily or oral dexamethasone at 0.3–0.6 mg/kg daily (or an equivalent dose of intravenous dexamethasone during inpatient stay), up to a maximum prednisolone dose of 60 mg/day in the first 4 weeks. The total course is 6–8 weeks, including gradual tapering of dose.

#### H2 receptor antagonist

Ranitidine prophylaxis is given for the first 8 weeks at 1 mg/kg three times per day (maximum per dose 3 mg/kg) for children aged 1–5 months, 2–4 mg/kg two times per day for those aged 6 months to 11.9 years (max 150 mg) and 300 mg at night-time for adolescents aged 12–18 years. If ranitidine is unavailable, cimetidine is used at 25–30 mg/kg/day in two divided doses for children aged ≥1 year and 20 mg/kg/day for children aged <1 year.

#### Pyridoxine supplementation

Pyridoxine (usually 5–12.5 mg/day) is given to children who are malnourished, adolescents and those receiving ART throughout treatment to prevent isoniazid-induced neuropathy.

### Study follow-up

Hospitalised participants are reviewed daily, with clinical progress, time to recovery from coma (Blantyre Coma Score) and other observations documented until discharge. All children return at weeks 4, 8, 16, 24, 36, 48 and 72 postrandomisation when ATT is dispensed, clinical assessments are performed, adherence and acceptability questionnaires are completed and AEs are assessed (online supplemental table S1b). Safety monitoring for toxicities using blood tests occurs at weeks 1, 2, 4 and 8 and at any other time points if clinically indicated. Clinical safety monitoring occurs at every follow-up visit. Total blood volumes and volumes required per study visit are considerably less than the blood volumes allowed for research in children internationally.^46^ Neurological function and neurocognitive assessment using mRS and Liverpool Outcome Score (LOS) are completed at weeks 24, 48 and 72. At these time points, urine is collected for pregnancy testing from all females >12 years of age or who have started menses, and HIV viral load testing is completed for children living with HIV.

### Functional outcome scoring

The paediatric mRS is used to assess gross motor function and has been recommended in a recent position paper on standardising TBM outcome measures.^47^ Additionally, the LOS—a simple screening questionnaire—is used to evaluate functional outcomes in all developmental domains. Neurological status and neurodevelopment at baseline are assessed with a short developmental history and completion of mRS and LOS on day 7 or at discharge, whichever is sooner. A full neurological examination is also completed at each follow-up.

### Quality of life questionnaires

Health outcomes are measured in quality-adjusted life years, with health-related quality of life measured using EQ-5D-Y, a generic health status instrument, which will be collected via proxy from a caregiver when the patient is cognitively or physically incapable (http://www.euroqol.org/eq-5d-products/eq-5d-y-youth.html). Data will be included in cost-effectiveness analyses in the health economics substudy (table 3).

**Table 3:** Overview of nested SURE substudies

Substudy	Objective	Study design	Sites
Diagnostic and prognostic biomarkers*	Discover novel CSF, serum, urinary diagnostic and prognostic biomarkers or signatures using omic technology	Nested case-control, which prospectively recruits children with symptoms and/or signs of CNS infection but does not have TBM	200 controls: Zimbabwe and Zambia (100) and Vietnam (100)
PK	Determine whether the doses of anti-TB drugs, prescribed according to weight bands, result in appropriate drug plasma and CSF exposures	Intensive PK (n=36) in the intervention arm—six per PK grouped weight band.Sparse PK (n=108)—36 from the intervention arm (6/grouped weight bands) and 72 from the control arm (12/grouped weight bands).One CSF sample time-matched with plasma.All samples were taken on day 14 after autoinduction of rifampicin had occurred.Non-compartmental PK techniques and NONMEM.Penetration of TB drugs across the blood-CSF barrier; predictors of exposure will be assessed and relationships between exposures achieved and response will be assessed (PK-PD modelling).	Intensive PK—VietnamSparse PK—all sites
Pharmacogenetic	Evaluate genetic differences in drug metabolic pathways affecting TB drug exposure, including NAT-2	Store DNA/cell pellets from whole blood taken at any time during the study.	All sites
MRI	Evaluate the impact of aspirin on brain ischaemic changes between baseline and 6 months	MRI was performed at two time points using predetermined and standardised sequencing.Three independent radiologists blinded to the treatment arm and clinical condition review each MRI.	Vietnam (50 participants) and India (minimum 50 participants)
In-depth neurodevelopmental and neurocognitive studies	Explore the effect of treatment on neurodevelopmental outcomes	BSID for children under 3 years of age.Kaufmann Assessment Battery for children aged 3–18 years at 48 and 72 weeks.Together with mRS and LOS, the impact of disease severity at presentation on developmental domains will be determined.	Vietnam
Health economics†	Acceptability and health economic evaluations are conducted in the trial to help inform policy-making and scale up the implementation of the shortened-intensified anti-TB drug therapy if it is found to be non-inferior	Cost-effectiveness on trial comparisons, with health outcomes measured in QALYs and costs estimated from alternative perspectives (healthcare, patient/family, societal).Decision analytic modelling techniques will be used to synthesise evidence within the trial, with external evidence, to estimate impacts over the lifetime of patients.	All (analysis by University of York)
Epilepsy	Characterise frequency, type of seizure in the acute phase and epilepsy at 18 months follow-up.Factors associated with the development of epilepsy will be explored.	Seizure history, including number of antiseizure medications taken during hospitalisation and 18-month follow-up.The severity of epilepsy is determined using E-CHESS.Electroclinical syndrome will be reviewed by 2 paediatric neurologists.	India

* The diagnostic substudy slightly differs in time points for sample collection and follow-up between Zambia, Zimbabwe and Vietnam.

† In addition to health economics analysis at all sites, a cost-of-illness and social impact of TBM will be conducted to determine the impact on patients/families using a ‘one-on-one’ interview approach in India.

BSIDBayley Scales of Infant DevelopmentCNScentral nervous systemCSFcerebrospinal fluidE-CHESSEarly Childhood Epilepsy Severity ScaleLOSLiverpool Outcome ScoremRSmodified Rankin ScaleNAT-2N-acetyltransferase type 2NONMEMnon-linear mixed effects modellingPDpharmacodynamicPKpharmacokineticQALYsquality-adjusted life yearsTBtuberculosisTBMtuberculous meningitis

### Nested substudies

A PK substudy is currently underway in selected African and Asian sites and will provide important data on drug exposure of all trial TB drugs (sourced from the same manufacturer) and their relationship with toxicity (eg, drug-induced liver injury (DILI)) and response. Pharmacogenetic substudies (eg, acetylator status) to evaluate genetic differences affecting exposure to TB drugs are planned. In-depth characterisation of longitudinal neuroimaging with MRI will assess the impact of aspirin on cerebral ischaemia/infarcts, as well as neurocognitive and epilepsy outcomes. Substudies on pathophysiology, epilepsy and health economic evaluations are also underway (table 3). SURE also has a multi-site nested substudy for the discovery of novel diagnostic and prognostic biomarkers in CSF and non-CSF clinical samples. Using a case-control design, controls for the diagnostic substudy are children who fail screening and have an alternative diagnosis. Embedding this design into the trial framework allows full use of all screened participants and sensitises parents/carers to lumbar punctures, which is beneficial in settings where lumbar puncture refusal is high. Diagnostic challenges are also opportunities for capacity strengthening (eg, lumbar puncture workshops and educational videos on consenting carers/parents to lumbar puncture).

### Serious AEs (SAEs) and safety reporting

The definitions in the European Union (EU) Directive 2001/20/EC, Article 2, based on the principles of Good Clinical Practice (GCP), apply to this trial protocol. Serious adverse reactions (ARs) include any untoward or unintended response to drugs.

All SAEs meeting the definitions as per the EU guidance (ENTR/CT 3, April 2006 revision) are reported regardless of their relationship to TBM. AEs and/or ARs (serious and non-serious) are graded using the toxicity gradings in the 2017 Division of AIDS (DAIDS) toxicity grading V.2.1.

An AE is deemed to be serious if it meets one of the pre-defined six criteria online supplemental table S5, row 4. The investigator provides a causality assessment of all serious events in relation to the trial therapy.

Where causality is assessed as unrelated or unlikely to be related to trial medication, the event is classified as an SAE. Where causality for SAE is assessed as possible, probable or definitely related to trial medication, the event is classified as a serious AR (SAR). SARs will also undergo an expectedness assessment by the MRCCTU Clinical Reviewer, and if deemed unexpected, the SAR is classified as a suspected unexpected SAR and requires expedited regulatory reporting.

All SAEs occurring from the time of randomisation until the participant’s final 72-week follow-up visit are reported. SARs are notified until trial closure.

### Events of interest

A number of events that could have an impact on drug management but would not be considered serious and therefore not constitute an SAE are reported as an Event of Interest (EOI) as in Box 2.

Box 2 **Event of interest**

- Grade 3/4 adverse event/reaction.
- Gastrointestinal bleeding (any grade).
- Drug-induced liver injury (grade 2 and above).
- New development of obstructive hydrocephalus.
- Suspected relapsed tuberculosis disease.
- Tendonitis or tendon pain (any grade).
- Muscle/joint aching/pain (grade 2 or above).

Pregnancy is reported as an EOI in an expedited manner. Pregnancy-related events do not constitute SAEs unless they result in a condition that meets the seriousness criteria defining SAEs (eg, septic abortion). All pregnancies are followed up until their outcome is known and recorded.

### Withdrawal of trial treatment

Protocol treatment discontinuation is considered under any of the following circumstances:

- Unacceptable toxicity or AE.
- Intercurrent illness that prevents further treatment.
- Any change in the patient’s condition that justifies the discontinuation of treatment in the clinician’s opinion.
- Withdrawal of consent for treatment by the patient or parents/carers of young children.
- Suspected treatment failure.
- Drug resistance (ATT-specific).

Children who have protocol treatment discontinued remain in the trial for the purpose of follow-up and data analysis (including up until the time a carer withdraws consent for follow-up if this occurs). Data are kept and included for patients who stop follow-up early, up to the point of exit.

### Patient and public involvement

Patients and public representatives who are survivors of TBM (from Vietnam and Zimbabwe) will be involved in the dissemination of study results to participants and linked communities. There was no specific patient and public engagement at the time of study design, protocol development or recruitment and retention, in part because of the global COVID-19 pandemic.

### Data handling and data management

Each site is responsible for its own data entry and local trial management. Data are entered into the trial database directly at the site and checked centrally by authorised personnel. Data are checked for missing or unusual values and consistency within participants over time. When problems are identified, sites are contacted to verify or correct data entries on the original case report form (CRF) and the database.

### Quality control (QC) and assurance

The quality assurance and QC considerations have been based on a formal risk assessment. This risk assessment has been reviewed by the MRCCTU’s research governance committee and has led to the development of a data management plan, safety reporting plan and monitoring plan, which are separately reviewed by the MRCCTU Quality Management Advisory Group.

### Monitoring

Staff from the relevant clinical trial unit (CTU) visit clinical sites to validate and monitor data, and when this is not practical, an independent local monitor makes regular monitoring visits to the trial sites. The frequency, type and intensity of routine on-site monitoring and the requirements for triggered monitoring are detailed in a monitoring plan. Central monitoring occurs at MRCCTU through regular database reviews and reviews by the trial statistician for missing or inconsistent data. Queries are raised to sites that are responsible for their resolution.

### Statistical considerations

#### Sample size

The study is powered by the comparison of the shortened ATT versus standard control ATT, as this requires a larger number of patients. Based on a non-inferiority margin of 10%, 394 (rounded to 400) children would be needed to demonstrate non-inferiority, with 90% power, 2-sided 5% alpha and 5% loss to follow-up at 1 year, assuming 20% mortality in the control regimen and 17% (ie, a 3% (absolute) improvement) in the intensified shorter regimen. Control arm estimates are based on a recent meta-analysis,^3^ and the assumption of a 3% improvement in the intervention arm is based on the reported lower mortality with the intensified short regimen used in Cape Town.^15^

Assuming 50% of children in the placebo arm die or suffer severe impairment,^3^ 400 children would provide approximately 90% power, 2-sided 5% alpha, to detect a difference of at least 16% (superiority margin) in the aspirin arm when compared with placebo. The largest TBM aspirin trial to date reported 45% and 50% reductions in stroke and mortality, respectively.^41^ Following regular consultations with the independent data monitoring committee (IDMC) and trial steering committee (TSC), if the planned recruitment of 400 children is not possible, the modified target of 300 children would be acceptable to allow 80% power and 350 children to allow 85% power.

#### Statistical analysis plan (SAP)

Analysis populations for both randomisations will be (1) intention to treat (ITT) population defined as all randomised children who took at least 1 dose of the trial drug (either ATT or aspirin), (2) modified ITT (mITT) defined as ITT excluding late screening failures (ie, those randomised but subsequently not found to have TBM) and (3) per-protocol population defined as mITT excluding those not taking at least 80% of allocated treatment (unless in case of death). The latter also includes enrolled participants who were later found to be ineligible due to a major protocol deviation affecting efficacy (eg, rifampicin resistance).

#### Primary analysis

Primary outcomes for *randomisation 1* is mortality from all causes at 48 weeks. The primary analysis is based on the difference in proportion of deaths between the two arms at 48 weeks. The difference will be adjusted for minimisation factors using Cochran-Mantel-Haenszel weights. Alternative methods will be considered should sparse data within strata lead to unstable estimation.

Non-inferiority will be assessed using the upper bound of the two-sided 95% CI for this absolute difference. If the upper bound of this CI is less than 10% (the margin of non-inferiority), the intervention arm is considered to be non-inferior to the control arm.

*Randomisation 2* is designed to test for superiority of the aspirin versus placebo. mRS outcomes will be dichotomised as death (score 6) or severe impairment (score 5) versus the rest (score 0–4) and will be analysed using logistic regression adjusting for the minimisation factors.

As a secondary analysis, mRS will also be analysed using four groups: death or severe impairment (scores 5–6), moderate disability (scores 3–4), mild disability (scores 1–2) and full recovery (score 0).

This factorial trial assumes no statistical interaction between the two randomisations. An mITT analysis will be considered primary, and any exclusion from a full ITT analysis is detailed and justified in the SAP.

#### Planned subgroup analysis

All subgroup analyses described here are exploratory. Subgroup analyses will be carried out and analysed for the primary efficacy endpoint for the mITT population. Subgroup analyses defined below will be performed for the mITT primary analysis for each randomisation 1 and 2 (Box 3).

Box 3 **Planned Subgroup analyses**

- Region* (Africa, India and Vietnam).
- Age group* (<2 years and ≥2 years).
- Sex (male and female).
- TBM stage using BMRC (1 and 2 combined, 3)*.
- ATT treatment prior to randomisation (< 7 days; ≥ 7 days)
- HIV status*.
- TBM baseline categorisation (definite and probable combined, possible).

*Denotes minimisation factor. ATT, antituberculosis therapy; BMRC, British Medical Research Council and TBM, tuberculous meningitis.

## Ethics and dissemination

Ethics approval was received from the University College London (UCL) research ethics committee, London, UK (14935/0010); the institutional ethics committee, Postgraduate Institute of Medical Education and Research, Chandigarh, India (IEC-04/2019–1171); the ethics committee for human research, Lady Hardinge Medical College, Delhi, India; the University of Zambia Biomedical Research ethics committee, Zambia (0050419); joint research ethics committee University of Zimbabwe, Zimbabwe (83/19); the Mulago Hospital research and ethics committee; the ethics research committee Pham Ngoc Thach Hospital, Ho Chi Minh City, Vietnam; the ethics research committee Children’s Hospital 2, Ho Chi Minh City, Vietnam; the ethics research committee National Lung and TB Hospital, Ha Noi Vietnam; the ethics research committee Vietnam National Children’s Hospital, Ha Noi Vietnam; the Oxford tropical research ethics committee and the ethics research committee of the Ministry of Health, Vietnam.

Written consent is sought from parents/guardians, and assent from children who are developmentally appropriate and age-appropriate (as per local guidance) prior to trial participation. In the event a child is unable to give assent, for example, due to an altered conscious state, assent is deferred until recovery, and parental consent is used for enrolment. Opinions of children on their participation in the trial are taken on board at every opportunity, including assessments and follow-up visits. The rights of the child and/or their carer to refuse participation in the trial without giving a reason are respected, and withdrawal can occur at any stage of the trial. Re-consent is taken for children who turn 18 years of age during the trial (online supplemental material).

During the consent process, study objectives, the voluntary nature of participation and different treatments and possible toxicities by arm are explained to carers/participants using patient information sheets and reinforced during visits. An opt-in and opt-out option is provided on consent forms for participation in substudies, including a separate consent form for the diagnostics substudy (online supplemental material). The 6-month intensified ATT regimen has shown to be efficacious and safe but has never been subjected to a randomised comparison. Equipoise exists for aspirin/placebo in TBM given the lack of conclusive evidence from clinical trials to date (in adults or children). Previous concerns regarding the risks of developing the rare Reye syndrome in children have not stood up to recent scrutiny, nor has it been evident when using high doses in Kawasaki disease.

Safe CSF volumes are collected following published guidance.^44^ Total blood volumes and volumes required per study visit are considerably less than the blood volumes allowed for research in children internationally.^46^

Prior to the initiation of the trial at each clinical site, the protocol, all informed consent forms and carer/patient information materials are submitted to the appropriate ethics committee for approval. Further protocol amendments will also require ethical approval before implementation.

The principles of the UK General Data Protection Regulation are followed at all participating sites. Participants’ anonymity is maintained, and identities are protected from unauthorised parties. Clinical information will not be released without written permission, except as necessary for monitoring purposes. All consent forms and CRFs are stored in a secure filing cabinet at study sites. Only the PI and local investigators have access to the trial dataset solely for research purposes.

The SURE TSC is the custodian of the data and specimens generated from the SURE trial. All publications will be approved by the Trial Management Group (TMG) and TSC before submission for publication. Any publication arising before the end of the trial (not by randomised groups) will also be approved by the IDMC. In line with UCL policy, the results of publicly funded research will be freely available, and manuscripts arising from the trial will, wherever possible, be submitted to peer-reviewed journals, which enable Open Access via UK PubMed Central within 6 months of the official date of final publication. Dissemination of findings will also occur with patient and public involvement (PPI) and community advisory board input to communities, media, international TB conferences and key stakeholders, including the WHO Child and Adolescent TB Working Group. All conference presentations will be made available as soon as possible after the event via the MRCCTU and participating trial site websites.

### Study status

Enrolment in the SURE trial commenced in March 2021, and trial follow-up will be completed by the end of December 2025. Recruitment of 369 children was completed in June 2024, at which time loss to follow-up was low.

### Indemnity

Participants exit the trial at 72 weeks without post-trial care. UCL holds insurance against claims from participants for injury caused by their participation in the clinical trial. In addition, research facilities/clinics have a duty of care to the participant and hold clinical negligence insurance cover for harm caused during the trial.

### Trial governance and committees

A TMG comprising the Chief Investigator, lead site investigators and members of the MRCCTU and Oxford University Clinical Research Unit Clinical Trial Unit (OUCRU CTU) teams is responsible for the day-to-day management of the trial. The IDMC is the only group that sees confidential, accumulating data for the trial by the randomised arm and can recommend premature closure or reporting of the trial to the TSC.

The TSC has membership from the TMG plus independent members, including the Chair. The TSC provides overall supervision for the trial and advice. The ultimate decision for the continuation of the trial lies with the TSC. The frequency of meetings is dictated in the IDMC and TSC charters but has been around every 6 months to date.

A central (blinded) endpoint review committee (ERC) will ascertain the primary endpoint for TBM in control versus intervention arms for both randomisations 1 and 2. Additional endpoints to be reviewed will be:

- All causes of death.
- Specific AEs, for example, DILI (grade 2 and above) and all SAEs.
- Prespecified secondary endpoints (online supplemental table S6).

Furthermore, the ERC will determine if the event is related to the underlying TBM disease or to the treatment.

UCL is the sponsor of SURE and delegates to MRCCTU and OUCRU the responsibility of overseeing the implementation of the study by ensuring that arrangements are put into place for adequate management, monitoring, analysis and reporting of the trial.

UCL is the SURE trial sponsor (MRCCTU at UCL, 90 High Holborn St., London).

## Discussion

Interventional trials in paediatric TBM are few and challenging to conduct but are critically important for improving the treatment of TBM, which disproportionately kills or disables children. The SURE trial is the largest randomised paediatric TBM treatment trial to date and provides an opportunity to address several other important research questions. It is the first phase-3 trial to study both the role of a shortened intensive ATT regimen and anti-inflammatory therapy for childhood TBM using a factorial design. Children bear the heaviest burden of TBM, yet this vulnerable group, which stands to benefit the most from new treatment approaches, is often excluded from randomised clinical trials (RCTs). Much of the mortality and morbidity is driven by the host immuno-inflammatory response to *M. tuberculosis*. Treatment advancements to reduce mortality and neurological sequelae from TBM should, therefore, evaluate both new anti-inflammatory agents and optimised ATT regimens, where possible in the same trial.

In recent years, there has been a surge in adult TBM treatment clinical trials, which primarily aim to optimise treatment by intensifying the ATT regimen with high-dose rifampicin (up to 35 mg/kg), with or without linezolid as an alternative fourth agent.^48^ However, most active trials exclude children and even adolescents (table 1) and to date, there has been just one other paediatric TBM ATT treatment trial, TBM-KIDS. In this phase 2 open-label trial, children were randomised to high-dose rifampicin (30 mg/kg), with or without levofloxacin (instead of ethambutol) for 8 weeks, followed by 10 months of continuation phase versus standard of care.^22^ However, due to challenges with recruitment, this trial was halted after just 37 children were randomised. While treatment shortening to 6 months for drug-susceptible TBM has been the standard of care in the Western Cape of South Africa for more than 30 years, and has informed a recent conditional treatment recommendation by the WHO,^49^ there have been no published head-to-head clinical trials with the standard of care. Two active TBM treatment-shortening trials are in progress, of which SURE is the only paediatric one. The other, IMAGINE-TBM (Improved management with antimicrobial agents isoniazid rifampicin linezolid for TBM) adult trial is a phase 2/3 randomised open-label trial comparing a 6-month TBM treatment regimen of high-dose rifampicin (35 mg/kg), high-dose isoniazid (10–15 mg/kg), pyrazinamide (25 mg/kg) and linezolid (1200 mg) for 8 weeks followed by rifampicin (35 mg/kg) and isoniazid (10 mg/kg) for 16 weeks (total 24 weeks) to a 9-month standard-of-care regimen in adolescents and adults. Unlike our trial, IMAGINE-TBM does not include adjuvant anti-inflammatory as an additional intervention. The additional advantage of the SURE trial design is its broad generalisability. If shortened, intensified ATT is non-inferior to the current 12-month standard, we anticipate swift transfer into clinical practice via changes to WHO guidelines, which are followed in most low-middle income countries. If high-dose aspirin is shown to be superior and safe, an inexpensive and widely accessible treatment would then be rapidly implemented into guidelines. Thus, the SURE trial is uniquely placed to answer important research gaps in the neglected field of TBM and potentially provide new TBM treatment approaches to improve outcomes while significantly reducing the duration of treatment. Study limitations include the enrolment of children who may not truly have TBM, as microbiological evidence of *M. tuberculosis* in CSF was not mandated for enrolment in this pragmatic trial, and mortality rates may not be an accurate reflection of the real world due to close monitoring and follow-up within the trial context.

## supplementary material

10.1136/bmjopen-2024-088543 online supplemental file 1

10.1136/bmjopen-2024-088543 online supplemental file 2
