# Supplementary material for: Effectiveness and safety of shortened intensive treatment for children with tuberculous meningitis (SURE): a protocol for a phase 3 randomised controlled trial evaluating 6 months of antituberculosis therapy and 8 weeks of aspirin in Asian and African children with tuberculous meningitis
Source: BMJ Open. 2025 Apr 2;15(4):e088543. doi: 10.1136/bmjopen-2024-088543 (PMC11967003; doi:10.1136/bmjopen-2024-088543)
Supplement: online supplemental file 1 [file bmjopen-15-4-s001.pdf]

INFORMATION SHEET FOR PARENTS OR CARERS

[To be presented on local headed paper]

[Insert: Date & Version]

SURE clinical trial: Short intensified treatment for children with tuberculous meningitis
(ISRCTN40829906)

Local Investigator: Dr. ……………………………………

We are inviting your child to take part in a research study

- Before you decide whether you would like your child to take part, it is important for you to understand why the research is being done and what it will involve
- Please take time to read the following information carefully and ask the study doctor or nurse if there is anything you do not understand or you would like more details
- You are free to decide if you want your child to take part in this research study. If you decide that you do not want your child to take part, it will not affect the care he/she receives. If your child joins the study you can later decide to withdraw him/her from the study at any time without giving a reason.
- If your child takes part in this study they will be seen by the study team every day while they are in hospital. After your child has been sent home (discharged) from hospital we will see them at the clinic regularly; 2, 4 and 8 weeks after starting treatment, then roughly every 3 months for a year and a final visit at 18 months to see how your child is recovering. We will refund your transport costs. Thank you for taking time to read this information. If you decide you would like your child to take part, we will ask you to complete a consent form and you will be given a copy of this information to keep.

Contents

1. Why are we doing this study?
2. Why have I been asked if my child can take part in the SURE study?
3. What do I need to know about the medicines in this study?
4. What will happen if my child takes part in the SURE study?
5. What are the possible side effects?
6. What are the possible benefits and disadvantages of taking part?
7. Storage of blood and other samples
8. More information about taking part
9. Contacts for further information

1. Why are we doing this study?

This study is for children with TB of the brain (TB meningitis).

a. What is TB meningitis?

TB meningitis is a serious infection of the brain caused by the tuberculosis (TB) germ. Children who spend time close to an adult who has TB may easily get TB themselves, and in young children this can spread to the brain and cause serious illness and long-term brain damage resulting in severe disability. It can be difficult to diagnose the infection and as a result children may be severely ill by the time they get treatment.

b. How is TB meningitis usually treated?

Children with TB meningitis have to take TB medicines every day for at least 12 months. They are also given a medicine (a steroid) that reduces swelling in the brain for the first 4 weeks of treatment.

c. What are we trying to find out?

Children with TB meningitis are usually given 12 months of TB medicines as currently advised by the World Health Organisation (WHO). We are testing whether giving children higher doses of these TB medicines, together with an extra TB medicine, for 6 months will treat TB meningitis just as well.

We also want to find out whether giving aspirin (a simple medicine often used to treat fever and thin the blood) during the first 2 months of TB treatment reduces the chance of children becoming disabled.

2. Why have I been asked if my child can take part in the SURE study?

Your child is being invited to join this study because he/she has been diagnosed with TB meningitis.

3. What do I need to know about the medicines in this study?

If your child takes part in this study he/she will either be given the TB medicines that are usually used to treat children with TB meningitis for 12 months (standard of care treatment) OR the higher dose TB medicines for 6 months (test treatment).

The medicines are:

- Standard of care treatment:
  4 medicines (rifampicin, isoniazid, pyrazinamide and ethambutol) daily for 2 months followed by 2 medicines (rifampicin and isoniazid) daily for 10 months.

  OR

- Test treatment:
  4 medicines (rifampicin, isoniazid, pyrazinamide and levofloxacin) daily for 6 months.

In addition your child will also need to take either:

- Aspirin daily for 2 months

  OR

- Placebo (a pill that looks just like aspirin but contains no medicine) daily for 2 months

Whilst they are taking the aspirin or placebo, your child will also be given a medicine called ranitidine to protect their stomach.

Your child will also receive steroid medication for the first 4 to 6 weeks of their TB meningitis treatment. This is standard of care for TB meningitis.

4. What will happen if my child takes part in the SURE study?

a. Screening

If the study doctor thinks your child has TB meningitis you will be asked to give your consent that your child can take part in the SURE study. Your child will be examined and routine tests completed to make the diagnosis and identify the TB germ in their body. These tests are described in section 4c.

The results of most of these tests will be ready on the same day and help the study doctor decide if your child has TB meningitis and also how sick your child is. The study doctor or nurse will discuss the results of these tests with you. Some of the special new tests for TB will take longer to come back.

If we find that your child is sick from any other illnesses or infections (at the beginning of the study and during the study) we will make sure that your child receives the right treatment.

b. Who decides what treatments my child will get?

Whether your child will get TB treatment for 6 months or 12 months and whether they will get aspirin or placebo pill (which contains no medicine) for 2 months, is chosen by a computer using a process called 'randomisation'. This will decide which one of the four groups of treatment your child will go into. It means your child will have an equal chance of being treated with TB medicines for 6 months or 12 months and of getting aspirin or placebo pill for 2 months. This makes sure that the groups of children being compared in the SURE study are as similar as possible, except for the different treatments they receive. There is no advantage of going into one treatment group over the other as they should all be at least as good as the current, WHO standard treatment for TB meningitis.

c. What tests will my child have?

Most of these tests are done for any child with TB Meningitis and are considered good practice.

- We will weigh your child, measure his/her height or length and the size of their head.
- **Lumbar puncture (spinal tap):** This is a safe procedure that is standard care for TB meningitis. It involves taking a small sample of cerebral spinal fluid (CSF), which is the fluid that surrounds the brain and spinal cord, for examination. A sample of up to 3 teaspoons of CSF, depending upon the age and size of your child, will be taken through a special needle in the lower part of your child's back. The CSF sample will be sent to a laboratory to look for the germs that cause TB meningitis and to check that the TB medicines will kill the germs.
- **Chest X-ray:** We will take an X-ray of your child's chest as TB can also affect the lungs and this may be seen on X-ray.
- **Sputum test:** For small children this might involve putting a tube through the child's nose and into the throat or the stomach to get the swallowed sputum. Older children may be asked to cough up sputum. We may give them a salty water spray through a mask to help them cough up sputum, if required. This is a commonly used method and is safe. The sputum will be tested in a laboratory to see if it shows TB germs.
- **Blood tests:** We will take a small amount of blood, up to 4 teaspoons, depending on the age and size of your child. The total amount of blood that we will take will never exceed a safe limit that

has been agreed by the authorities. We will use some of this blood to check for other germs that could be causing your child's illness, that the dose of medicine given is correct and to make sure that there are no problems caused by taking the medicines (like the liver or kidneys not working well). Your child's HIV status will also be checked, if it is unknown, as this could mean that extra HIV treatment is needed. Study staff will tell you the results of the HIV test. We will ensure your child gets HIV treatment if the test is positive.

- **Urine sample:** We will store a small sample of urine (about a teaspoon) to use on a new urine test for TB.
- **Neurocognitive tests (tests to check your child's development):** We will use questionnaires to see if your child's development (e.g. walking, talking, playing and learning) was normal before the illness and to see if it has been affected by the illness. Some children may be asked to complete some play based tasks to check their development more thoroughly.
- **[Brain scan:** We will take take pictures of your child's brain at the start of treatment and 24 weeks later to see how the brain images change or improve with treatment, this is one of the usual investigations for anyone with tuberculosis infection of the brain. If you do not wish to take part in the study you can decline to do so by not checking the checkbox on the consent form.]

d. Getting started

If the test results show that your child can take the TB meningitis medicines, and you agree to them joining the SURE study, they will start either the standard of care treatment (12 months of medicines) or the test treatment (6 months of medicines). Your child will usually stay in hospital for at least 7 to 14 days or until they are well enough to eat and drink and take medicines by mouth.

e. What happens after my child is discharged from hospital?

Once your child is well enough to go home you will be given enough medicines to give them every day until they come back to the clinic for the planned study visits. The study doctor and nurses will tell you all about these medicines and how to give them to your child.

Someone from the SURE study team may visit you and your child at home so that they can help remind you about planned study appointments and make sure that all is well if you are unable to bring your child for a study appointment as planned. If they cannot visit you at home they will remain in contact by telephone.

You will need to bring your child back regularly so that he or she can be checked, and to be given more medicines. The study nurses will help to ensure that you do not run out of medicines.

We know that it can be difficult giving medicines to your child every day. It is very important that:

- your child does not miss any doses of the TB meningitis medicines
- the medicines are not shared with anyone else

If your child does not take the TB meningitis medicines regularly they may not work properly.

We want to know how well children take their medicines and if there are any problems taking them. From time to time during the study we will ask you and your child some questions about their experiences of taking these medicines.

You should ask the SURE study team's advice before your child takes any other medicines.

Depending on which treatment group your child is in, after 6 or 12 months your child will stop taking the TB meningitis medicines, but will continue to come to the clinic for check-ups every 3 months until 18 months from the time your child started the TB meningitis treatment.

We will reimburse costs for transport to get you and your child to the study clinic and back to your home.

5. What are the possible side effects?

The medicines being used in the SURE study are all recommended by the WHO and are routinely used for treating adults and children in your country for TB meningitis. Although these medicines are generally safe in children, side effects may rarely occur.

The most common side effects caused by the TB meningitis medicines are orange tears and urine. This is not harmful at all.

Less commonly, the following side effects may occur:

- Joint pains
- Yellowing of the skin and eyes
- Itching skin and rash
- Vomiting or diarrhoea
- Problems with vision (very rare)
- Both aspirin and steroid medicine may cause the lining of the stomach to become irritated and bleed resulting in blood stained vomits or dark bowel motions. This should be prevented by taking the ranitidine medicine.

Please tell the study staff as soon as possible if you become concerned about any side effects. It may be necessary to stop the medicine until the problem goes away or it is safe to re- start the medicine. We will look for side effects carefully and replace any TB meningitis medicines that cause problems.

Blood collection: There are very few risks from collecting blood but side effects may include discomfort, bruising and (very rarely) infection. The study staff will make sure that the amount of blood taken is safe for your child.

Lumbar Puncture: The risks of lumbar puncture are also few and include discomfort when the needle is inserted into the back, the possibility of infection and headache.

6. What are the possible benefits and disadvantages of taking part?

a. What are the possible benefits of taking part in this study?

There may not be a direct benefit to your child from taking part in the SURE study apart from being seen regularly by a study doctor while they are in the study and being referred for further care, if needed. However, your child may benefit from taking treatment for a shorter length of time and also from improved recovery from TB meningitis by taking aspirin. The information we get from this study will also help us to improve future treatments for children with TB meningitis.

b. What are the possible disadvantages and risks of taking part in this study?

Your child needs to be treated for TB meningitis regardless of whether or not they join the SURE study. Your child might experience some side effects from the medicines that he/she takes in this study. Some of these could happen if your child took these medicines even if they were not in the

SURE study. Possible side effects of the TB medicines being used in the SURE study are listed above in section 5.

Your child will need to attend the study clinic more often than he or she would do if they were treated for TB Meningitis outside of the SURE study. He/she will also need to have additional blood tests.

It is important that you understand that if your child joins the SURE study, he/she will be in a research study which aims to find out whether taking a new combination of TB medicines for 6 months works as well as the standard 12 months of TB medicines. The medicines used in the SURE study are all recommended by WHO, and all are in use to treat TB. However, they may not all be the same as those currently used in your country's national TB treatment programme.

c. What about risks to pregnancy?

For girls who are 12 years old or above, or who have started to menstruate (started their monthly periods) a pregnancy test will be carried out before entry to the study and at regular intervals during the study.

Girls should avoid getting pregnant while in the study and use effective contraceptives if they are sexually active. Some of the study drugs will reduce the effectiveness of hormonal birth control and so barrier methods of contraception (e.g. condoms) should be used.

If girls do become pregnant they can continue to be part of the SURE study and receive the TB meningitis medicines. Once the baby is delivered, they will be assessed by a doctor and it maybe necessary to collect some health information about the baby.

d. What if my child has HIV?

Your child can still join the SURE study if he or she is has HIV.

7. Storage of blood and other samples

At some of the visits to the SURE clinic we may want to store some of the blood that was drawn so that special tests can be done later. This means that we may not give you the results of these tests at the time of your child's illness. We may also wish to store samples of your child's CSF and urine for future tests.

New and better tests to diagnose TB and TB meningitis are being developed and we are asking your permission to use your child's samples to see how well these new tests work compared to existing ones. This includes tests to look at how your child's genes determine whether they are more likely to develop TB meningitis than another child and why the TB medicines work more effectively in some children than others. Genes are found in every part of the body, and passed on from mother and father to their children. They control the way we look as well as the way the body responds to different illnesses.

These genetic and other tests might be done at some point in the future and may be done in or outside your country. The tests will be decided by a group of experts. The samples taken from your child will never be identified by their name and only approved people working on the study will have access to them.

You will not be given the results of findings from this genetic testing. However, any overall findings related to the presence of new genetic changes that may affect TB meningitis disease or treatment will be made available to the scientific, clinical and local community in order to improve the care and treatment of TB meningitis patients in future.

Your study nurse or doctor will be able to give you more information about this.

8. More information about taking part

a. Do I have to agree that my child can take part in the SURE study?

No, it is up to you to decide whether or not your child can take part. If you decide to allow your child to take part we will ask you to sign a consent form.

Your child can stop taking part in the study at any time. You won't have to give a reason. You can also decide that your child won't take part. This will not affect the care your child receives for their illness.

b. Who is organising the study?

UCL is the sponsor for this study is organised by the Medical Research Council Clinical Trials Unit at UCL (MRC CTU), which is based in the UK and which has run studies of this kind for many years. MRC CTU will manage the study and will collect and analyse the information.

Research institutions in India, Vietnam, Uganda, Zambia and Zimbabwe are conducting the SURE study.

c. Who has reviewed the SURE study?

The study has been reviewed by international TB meningitis experts as well as by *[insert name of local Research Ethics Committee].*

d. What will happen to information about my child collected during the study?

If your child takes part in the SURE study the doctors and nurses will collect information in accordance with our instructions whenever they see your child. University College London (UCL) will be using information from your child's medical records in order to undertake this study and will act as data controller for this study. The information will be put into a computer and sent to MRC CTU and will be analysed by SURE researchers. University College London (UCL), through the MRC CTU, is responsible for looking after your child's information and using it properly will keep identifiable information about you for 25 years after the study has finished.

The hospital will use your and your child's name, and contact details to contact you about the research study, and make sure that relevant information about the study is recorded for your child's care, and to oversee the quality of the study. UCL will collect information about your child for this research study from your hospital/clinic. This information will include health information, which is regarded as a special category of information. We will use this information to conduct our research.

We will follow all the laws and country specific data protection regulations as required to make sure that all information, including your child's identity and any personal details, will be kept confidential. No named information about you or your child will be published in any report of this study.

Your child's clinic notes may be looked at by study staff from UCL, relevant international regulatory authorities, the trials units co-ordinating the study or other authorised independent individuals to make sure that the SURE study is being properly carried out and to check the accuracy of the research study. They may also be seen by the companies who are supplying medicine for the study. Confidentiality will be maintained at all times.

We won't share information with others that can identify your child. The information will only be used for the purpose of health and care research, and cannot be used to contact you or to affect your child's care. It will not be used to make decisions about future services available to your child.

For more information about UCL privacy notice, please visit:

https://www.ucl.ac.uk/legal-services/privacy/participants-health-and-care-research-privacy-notice

e. What will happen to the results of the SURE study?

We will publish a summary of the results in a medical journal. You can ask the study staff for a copy of any publication.

Results will also be discussed in community meetings and with TB meningitis and HIV programme personnel.

f. What if new information becomes available during the SURE study?

Sometimes during a study new information becomes available about the medicines that are being used. An independent committee will look at any new information and will also look at the information collected during the study and decide if any changes are needed. If this happens, your doctor will tell you about it and discuss whether you want your child to continue in the study. If you decide to stop taking part in the study your doctor will arrange for your child's care and TB treatment to continue.

g. What if something goes wrong?

If you have any concerns about the way you or your child has been approached or treated during the SURE study, or wish to complain, please talk to the study doctors or nurses.

We do not expect that your child will be injured as a result of taking part in this study. However, in the unlikely event of injury resulting from your child's participation in this study please contact the study coordinator on the following telephone number: *[insert number]*

The *[insert name of local IRB]* oversees the conduct of this research study which is governed by the *[insert ethical guidelines governing the study].* If you have questions about your rights or your child's rights as a volunteer, you can also contact the *[insert name of local IRB]* through its chairman *[insert name]* on telephone number *[insert number].*

h. What about future research?

When you agree for your child to take part in a research study, the information about your child's health and care may be provided to researchers running other research studies in this organisation and in other organisations. These organisations may be universities, or companies involved in health and care research in this country or abroad. Your child's information will only be used by organisations and researchers to conduct research in accordance with relevant legislation, ethics and research policy requirements.

We won't share information with others that can identify your child. The information will only be used for the purpose of health and care research, and cannot be used to contact you or to affect your child's care. It will not be used to make decisions about future services available to your child, such as insurance. If there is a risk that your child can be identified the data will only be used in research that has been independently reviewed by an ethics committee.

9. Contacts for further information

If you want further information about the study, contact your study doctor, nurse or counsellor (see below).

[Insert address and telephone number of study doctor and/or nurse]

Contact:_________________________

Tel:_____________________________

Thank you for taking the time to consider taking part in the SURE study.

(To be presented on local-headed paper)
(insert version no and date)
Informed Consent Form - Parents and Carers

Study number:

SURE: Short intensified treatment for children with tuberculous meningitis

Please initial (or mark) each box if you agree

1. I confirm that I have read (or someone has read and explained to me) the information sheet *(insert version no and date)* for the SURE study and that I understand what will be required if my child participates in the study. The study has been explained to me and my questions have been answered.	
2. I understand the SURE study will involve my child being seen regularly by the doctor who will ask questions about my child's health, and my child having some samples taken for testing, including an HIV test, a chest X-ray, lumbar puncture and other routine TB meningitis tests as clinically indicated.	
3. I understand that my child will be given TB meningitis treatment at the recommended doses for 6 months or 12 months while he or she participates in the SURE study.	
4. I understand that my child will be given aspirin or placebo (a pill that contains no medicine) for the first 2 months of their treatment.	
5. I understand that my child's participation in all aspects of this study is voluntary and that I am free to withdraw my child from the study at any time, without giving any reason and without his/her medical care or legal rights being affected.	
6. I understand that sections of any of my child's medical notes may be looked at by responsible individuals involved in the running of the SURE study or from regulatory authorities where it is relevant to my child's participation in this research. I give permission for these individuals to have access to my child's records, but understand that strict confidentiality will be maintained.	
7. I understand if I am unable to continue to be the main carer for my child that I need to provide the SURE study with the name of the person who will become the main carer so their consent can be requested.	
8. I agree to allow samples to be taken from my child to be stored for later testing, including genetics testing. I understand that these samples will not be identified by either my or my child's name and that I will not be given results of findings from these tests relating directly to my child, but that the overall results may be used to improve the care and treatment of TB meningitis patients in future.	
9. I agree to allow my child to have brain images taken (a MRI scan) and the data being stored and used as part of the SURE study.	
10. I agree to allow the SURE care team to try and obtain information about my child's wellbeing from their clinic or hospital notes and the TB register at the end of the study (when the last participant enrolled has been in the study for 18 months).	
11. I agree that all information collected on my child during the trial can be made available to others in the future (open access) provided no one can identify my child from the details provided.	

12. I agree to my child taking part in the SURE study which is assessing whether it is safe and effective to have 6 months of TB meningitis treatment compared to the standard of 12 months of treatment.	

Parent/carer's signature (or thumbprint)	Print name	Date

Witness's signature (if thumbprint above)	Print name	Date

I have provided the SURE study information to the participant's parent/legal guardian in full and answered all his/her questions. To the best of my knowledge, he/she understands the purpose, interventions, risks and benefits of this study and willingly agrees for his/her child to be enrolled into the SURE study.		
Signature of person conducting the informed consent process	Print name	Date

IMPORTANT: one signed original to be kept in SURE investigator site file by the researcher
one signed copy to be given to the patient
one signed copy to be kept in the participant's clinic file

INFORMATION SHEET FOR PARTICIPANTS ABLE TO GIVE ASSENT

[To be presented on local headed paper]

[Insert: Date & Version]

SURE clinical trial: Short intensified treatment for children with tuberculous meningitis
(ISRCTN40829906)

Local Investigator: Dr. ……………………………………

We are inviting you to take part in a research study

- Before you decide whether to take part, it is important for you to understand why we want to carry out this study and what it will mean for you.
- Please take time to read the following information carefully and ask the study doctor or nurse if there is anything you do not understand or you would like to know more.
- You are free to decide if you want to take part in this research study. If you choose not to take part, it will not affect the care you receive.
- If you join the study you can stop taking part at any time without giving a reason.
- Thank you for reading this information, this copy is for you to keep.

Contents

1. Why are we doing this study?
2. Why have I been asked to take part in the SURE study?
3. What do I need to know about the medicines in this study?
4. What will happen if I take part in the SURE study?
5. What are the possible side effects?
6. What are the possible benefits and disadvantages of taking part?
7. Storage of blood and other samples
8. More information about taking part
9. Contacts for further information

1. Why are we doing this study?

This study is for children with tuberculous meningitis (TB meningitis).

a. What is TB meningitis?

TB meningitis is an infection of the brain caused by the TB germ. Children who spend time close to an adult who has TB may easily get TB and in children this can spread to the brain and cause serious illness.

b. How is TB meningitis usually treated?

Children with TB meningitis have to take medicines every day for at least one year. They are also given an extra medicine for the first month.

c. What are we trying to find out?

We are testing whether giving children a different combination of medicines for 6 months will treat TB meningitis as well as when giving them the standard medicines for a year.

We also want to find out whether giving aspirin, a simple medicine often used to treat fever, during the first 2 months of treatment helps children recover better from TB meningitis and reduces the risk of them becoming disabled.

2. Why have I been asked to take part in the SURE study?

You have been invited to join this study because you have been diagnosed with TB meningitis.

3. What do I need to know about the medicines in this study?

If you take part in this study you will either be given:

- Medicines that are usually used to treat children like you with TB meningitis for 12 months: 4 medicines (rifampicin, isoniazid, pyrazinamide and ethambutol) daily for 2 months followed by 2 medicines (rifampicin and isoniazid) daily for 10 months

  OR

- the alternative combination of medicines for 6 months: 4 medicines (rifampicin, isoniazid, pyrazinamide and levofloxacin) daily for 6 months (some of these medicines are at higher doses than currently used in children with severe TB)

As well as the TB medicines you will also need to take either:

- Aspirin daily for the first 2 months

  OR

- A placebo pill that looks just like aspirin but contains no medicine daily for the first 2 months

Whilst you are taking the aspirin or the placebo pill (which contains no medicine), you will also be given a medicine, ranitidine, to protect your stomach.

You will also receive another medicine, called a steroid, for around the first 4 weeks of your treatment. This is usual treatment for TB meningitis.

4. What will happen if you take part in the SURE study?

a. Screening

If the study doctor thinks that you have TB meningitis you will be asked to sign an assent form to show that you agree to take part in the SURE study. Your parent/guardian will be asked to sign a consent form to show you have both understood the information.

You will be examined by a doctor who will do some further tests to make sure you have TB meningitis. These tests are described in section 4c.

The results of most of these tests will be ready on the same day and will help the doctor find out if you have TB meningitis.

If we find that you are sick from any other illnesses or infections (at the beginning of the study and during the study) your doctor will make sure that you receive the right treatment.

If the results show that you are not able to join the SURE study, the study team will talk to you and your carer about whether you need to start TB meningitis treatment.

b. Getting started

If the results show that you can take the TB meningitis medicines, and you and your carer have agreed that you can join the SURE study, we will begin your medicines and tests. A computer program will choose which treatment you will take so it is fair and we can truly compare the treatment groups. Neither you nor your doctor will be able to choose which group you are in. You will either receive the standard TB treatment for one year or the new TB treatment 6 months, together with either aspirin or a placebo pill (containing no medicine) for the first 2 months.

You will be admitted to hospital for treatment of TB meningitis and will usually stay in hospital for at least 7 to 14 days or until you are well enough to eat and drink and take medicines by mouth.

c. What tests will I have?

Most of these tests are done for any child with TB Meningitis and are considered good practice.

- We will weigh you and measure your height and the size of your head.
- **Lumbar puncture:** This procedure involves taking a small sample of the fluid that surrounds the brain and spinal cord, through a special needle in your back. We will take up to 3 teaspoons (15mls) of fluid, depending upon your age and size. The sample will be sent to a laboratory to look for the germs that cause TB meningitis.
- **Chest X-ray:** We will take an X-ray of your chest as TB can also affect the lungs and this may be seen on X-ray.
- **Sputum test:** Smaller children may have a tube put in their nose and into the throat or the stomach to get the swallowed sputum. Older children may be asked to cough up sputum. We may give you a salty water spray through a mask to help you cough up sputum, if required. This method is safe and is used as standard practice. The sputum is tested in a laboratory to see if it shows TB germs.
- **Blood tests:** We will take a small amount of blood, up to 3 teaspoons (15mls), depending upon your age and size. The total amount of blood taken will never exceed a safe limit. We will use some of this blood to check for other germs that could be causing your illness.
- **Urine sample:** We will collect and store a small sample of urine (about half a teaspoon) to use on a new urine test for TB.
- **Neurocognitive tests (tests to check your development):** We will use questionnaires to see if your development (e.g. walking, talking, playing and learning) was OK before the illness and to see if it has been affected by the illness. Some children may be asked to complete some play based tasks to check their development more thoroughly.
- **[Brain scan:** We will take take pictures of your brain to see how the brain images change or improve with treatment, this is one of the usual investigations for anyone with tuberculosis infection of the brain. If you do not wish to take part in the study you can decline to do so by not checking the checkbox on the consent form.]

d. What happens after I leave hospital?

Once you are well enough to go home you will be given enough medicine to take every day until you come back to the clinic. The doctor and nurses will tell you all about these medicines and how to take them.

After going home from hospital, you will need to come back regularly; 2, 4 and 8 weeks after starting treatment, then roughly every 3 months. These visits are so we can make sure you are OK and we can give you more medicines.

We know that it can be difficult taking medicines every day. We want to know how well children take their medicines and if there are any problems taking them. From time to time during the study we will ask you some questions about your experiences of taking these medicines.

Depending on which group you are in, after 6 or 12 months you will stop taking the TB meningitis medicines, but will continue to come to the hospital for check-ups every 3 months until 18 months from the time you started the TB meningitis treatment.

e. Taking TB Meningitis medicines regularly

It is very important that:

- you do not miss any doses of the TB meningitis medicines
- the medicines are not shared with anyone else

If you do not take the TB meningitis medicines as the study team tells you, they may not work properly.

5. What are the possible side effects?

The medicines being used in the SURE study are routinely used for treating adults and children in your country for TB meningitis. Although these medicines are generally safe, side effects may occur. The most common side effects caused by the TB meningitis medicines are orange tears and urine. This is not harmful at all.

Less commonly, the following side effects may occur:

- Joint pains
- Yellow skin and eyes
- Itching skin and rash
- Vomiting or diarrhoea
- Problems with vision (very rare)
- Both aspirin and steroid medicine may cause stomach ache.

Please tell your parent, carer or your nurse or doctor as soon as possible if you are worried about any side effects. It may be necessary to stop the medicine until the problem goes away or it is safe to re-start the medicine.

Taking Blood: There are very few risks from taking blood samples but side effects may include discomfort, bruising and (rarely) infection. The study staff will make sure that the amount of blood taken is safe for you.

Lumbar Puncture: The risks of lumbar puncture are also few but it might be uncomfortable when the needle is inserted into your back. We will ask you to lie down for a few hours after the test and make sure that you get enough fluids.

6. What are the possible benefits and disadvantages of taking part?

a. What are the possible benefits of taking part in this study?

We cannot promise that participating in the study will directly help you apart from being seen regularly by a doctor while you are in the study and being referred for further care, if needed.

However, you may benefit from taking treatment for a shorter length of time and you may also have a better recovery from TB meningitis if you are taking aspirin.

The information we get from this study will also help us to improve future treatments for children with TB meningitis.

b. What are the possible disadvantages and risks of taking part in this study?

You need to be treated for TB meningitis regardless of whether or not you join the SURE study. You might experience side effects from the medicines that you take in this study.

You will need to attend for more clinic visits than you would do if you were treated for TB meningitis outside the SURE study. You will also need to have extra blood tests.

It is important that you understand that if you join the SURE study, you will be in a research study which aims to find out whether taking the 6 month medicines works as well as the standard 1 year medicines. The medicines used in this research study are not new, are all recommended by experts and all are in use to treat TB. However, they may not all be the same as those currently used in your country's national TB treatment programme.

c. What about risks to pregnancy?

For girls who are 12 years old or above, or who have started their monthly periods a pregnancy test will be carried out before entering the study and at regular intervals during the study.

Girls should avoid getting pregnant while in the study and use effective contraceptives if they are sexually active. Some of the study drugs will reduce the effectiveness of hormonal birth control and so barrier methods of contraception (e.g. condoms) should be used.

If girls do become pregnant they can continue to be part of the SURE study and to receive the TB meningitis medicines. Once the baby is delivered, they will be assessed by a doctor and it maybe necessary to collect some health information about the baby.

d. What if you are living with HIV?

You can still join the SURE study if you are living with HIV.

7. Storage of blood and other samples

At some of the visits to the SURE clinic we may want to store some of the blood that was drawn so that special tests can be done later. We may also wish to store samples of your spinal fluid and urine for future tests.

Your study nurse or doctor will be able to give you more information about this.

8. More information about taking part

a. Do I have to agree that I will take part in the SURE study?

No, it is up to you to decide whether or not you take part. This will not affect the care you receive in any way.

You can stop taking part in the study at any time and you won't have to give a reason.

b. What will happen to information about me collected during the study?

If you take part in the SURE study, the doctors and nurses will collect information whenever they see you. The information will be put into a computer and will be analysed by SURE researchers. The people who analyse the information will not be able to identify you and will not be able to find out your name or contact details.

Your doctor and nurses will keep your name and contact details confidential and will not pass this information to anyone outside of the clinic. They will use this information as needed, to contact you

about the research study, to make sure that relevant information about the study is recorded for your care, and to oversee the quality of the study.

UCL is responsible for looking after your information and using it properly. We will follow all the laws as required to make sure that all information, including your identity and any personal details, will be kept confidential. Some of your medical information might be looked at by other authorised independent people to ensure that the SURE study is being properly carried out, but we won't share information with others that can identify you.

If the results of this study are reported or published your name will not be used nor any information that could be used to identify you. The study will be conducted in agreement with the country specific data protection regulations.

For more information about UCL privacy notice, please visit:
https://www.ucl.ac.uk/legal-services/privacy/participants-health-and-care-research-privacy-notice

c. What if new information becomes available during the course of the study?

Sometimes during a study new information becomes available about the medicines that are being used. An independent committee will look at any new information and will also look at the information collected during the study and decide if any changes are needed. If this happens, your doctor will tell you about it and discuss whether you want to continue in the study. If you decide to stop taking part in the study your doctor will arrange for your care and TB treatment to continue.

9. Contacts for further information

If you want further information about the study, contact your study doctor, nurse or counsellor (see below).

[Insert address and telephone number of study doctor and/or nurse]

Contact:_________________________

Tel:______________________________

Thank you for taking the time to consider taking part in the SURE study.

PARTICIPANT - ASSENT TO SURE STUDY

(To be printed on local-headed paper)
(Insert version no and date)

Study number:

SURE**: Short intensified treatment for children with tuberculous meningitis**

Please tick yes or no to the questions below

1. Have you read (or had read to you) the information about this study?	YES ☐ NO ☐
2. Do you understand what the study is about?	YES ☐ NO ☐
3. Have you asked all the questions you want?	YES ☐ NO ☐
4. Have your questions been answered in a way you understand?	YES ☐ NO ☐
5. Can we take some blood from you at each clinic visit?	YES ☐ NO ☐
6. Can we perform a lumbar puncture on you?	YES ☐ NO ☐
7. Do you understand the risks and benefits of taking part in the study?	YES ☐ NO ☐
8. Do you understand that you are free to stop the study at any time?	YES ☐ NO ☐
9. Do you agree to have brain images taken?	YES ☐ NO ☐
10. Do you understand that, if you take part in the study, a computer will decide what medicines you will take?	YES ☐ NO ☐
11. Are you happy to take part?	YES ☐ NO ☐

If any answers are **‘no’** or you don’t want to take part, **don’t sign your name**.
If you **do** want to take part, you can sign your name below.

Participant’s signature (or thumbprint)	Print name	Date
Witness’ signature (if thumbprint used above)	Print name	Date

The person who explained the study to you needs to sign too:

<table>
<tr><td colspan="3">I have provided the SURE study information to the participant in full and answered all his/her questions. To the best of my knowledge, he/she understands the purpose, interventions, risks and benefits of this study and willingly agrees to participate in the SURE study.</td></tr>
<tr><td>Signature of person conducting the informed consent process</td><td>Print name</td><td>Date</td></tr>
<tr><td></td><td></td><td></td></tr>
</table>

IMPORTANT: one signed original to be kept in SURE trial file by the researcher
one signed copy to be given to the participant
one signed copy to be kept in the clinic file

INFORMATION SHEET FOR PARTICIPANTS ABLE TO GIVE CONSENT

[To be presented on local headed paper]

[Insert: Date & Version]

SURE clinical trial: Short intensified treatment for children with tuberculous meningitis
(ISRCTN40829906)

Local Investigator: Dr. ……………………………………

We are inviting you to take part in a research study

- Before you decide whether to take part, it is important for you to understand why we want to carry out this study and what it will mean for you.
- Please take time to read the following information carefully and ask the study doctor or nurse if there is anything you do not understand or you would like to know more.
- You are free to decide if you want to take part in this research study. If you choose not to take part, it will not affect the care you receive. If you join the study you can later decide to withdraw from the study at any time without giving a reason.
- If you take part in this study you will be seen by the study team every day while you are in hospital. After you have been sent home (discharged) from hospital we will see you at the clinic regularly; 2, 4 and 8 weeks after starting treatment, then roughly every 3 months for a year and a final visit at 18 months to see how you are recovering. We will refund your transport costs. Thank you for taking time to read this information. If you decide you would like to take part, we will ask you to complete a consent form and you will be given a copy of this information to keep.

Contents

10. Why are we doing this study?
11. Why have I been asked to take part in the SURE study?
12. What do I need to know about the medicines in this study?
13. What will happen if I take part in the SURE study?
14. What are the possible side effects?
15. What are the possible benefits and disadvantages of taking part?
16. Storage of blood and other samples
17. More information about taking part
18. Contacts for further information

10.Why are we doing this study?

This study is for children with tuberculous meningitis (TB meningitis).

d. What is TB meningitis?

TB meningitis is an infection of the brain caused by the TB germ. Children who spend time close to an adult who has TB may easily get TB themselves and in children this can spread to the brain and

cause serious illness and long-term brain damage resulting in severe disability. It can be difficult to diagnose the infection and as a result patients may be severely ill by the time they get treatment.

e. How is TB meningitis usually treated?

Children with TB meningitis have to take medicines every day for at least one year. They are also given an extra medicine (a steroid) that reduces swelling in the brain for the first 4 weeks of treatment.

f. What are we trying to find out?

Children with TB meningitis are usually given 12 months of TB medicines as currently advised by the World Health Organisation (WHO). We are testing whether giving children higher doses of these TB medicines, together with an extra TB medicine, for 6 months will treat TB meningitis just as well.

We also want to find out whether giving aspirin, a simple medicine often used to treat fever and thin the blood, during the first 2 months of TB treatment helps reduces the chance of becoming disabled.

11. Why have I been asked to take part in the SURE study?

You have been invited to join this study because you have been diagnosed with TB meningitis.

12. What do I need to know about the medicines in this study?

If you take part in this study you will either be given the TB medicines that are usually used to treat children with TB meningitis for 12 months (standard of care treatment) OR the higher dose TB medicines for 6 months (test treatment):

- Medicines that are usually used to treat children with TB meningitis for 12 months:
  4 medicines (rifampicin, isoniazid, pyrazinamide and ethambutol) daily for 2 months followed by 2 medicines (rifampicin and isoniazid) daily for 10 months

  OR

- the alternative combination of medicines for 6 months:
  4 medicines (rifampicin, isoniazid, pyrazinamide and levofloxacin) daily for 6 months
  (some of these medicines are at higher doses than currently used in children with severe TB)

As well as the TB medicines you will also need to take either:

- Aspirin daily for the first 2 months

  OR

- A placebo pill that looks just like aspirin but contains no medicine daily for the first 2 months

Whilst you are taking the aspirin or the placebo pill (which contains no medicine), you will also be given a medicine, ranitidine, to protect your stomach.

You will also receive another medicine, called a steroid, for around the first 4 to 6 weeks of your treatment. This is standard of care treatment for TB meningitis.

13. What will happen if you take part in the SURE study?

f. Screening

If the study doctor thinks that you have TB meningitis you will be asked to sign an consent form to show that you agree to take part in the SURE study and show you have understood the information.

You will be examined by a doctor who will do some further tests to make sure you have TB meningitis. These tests are described in section 4c.

The results of most of these tests will be ready on the same day and will help the doctor find out if you have TB meningitis. The study doctor or nurse will discuss the results of these tests with you. Some of the special new tests for TB will take longer to come back.

If we find that you are sick from any other illnesses or infections (at the beginning of the study and during the study) your doctor will make sure that you receive the right treatment.

If the results show that you are not able to join the SURE study, the study team will talk to you about whether you need to start TB meningitis treatment.

g. Who decides what treatments my child will get?

Whether you will get TB treatment for 6 months or 12 months and whether you will get aspirin or placebo pill (which contains no medicine) for 2 months, is chosen by a computer using a process called 'randomisation'. This will decide which one of the four groups of treatment you will go into. It means you will have an equal chance of being treated with TB medicines for 6 months or 12 months and of getting aspirin or placebo pill for 2 months. This makes sure that the groups of children being compared in the SURE study are as similar as possible, except for the different treatments they receive. There is no advantage of going into one treatment group over the other as they should all be at least as good as the current, WHO standard treatment for TB meningitis.

h. What tests will you have?

Most of these tests are done for any child with TB Meningitis and are considered good practice.

- We will weigh you, measure your height or length and the size of your head.
- **Lumbar puncture (spinal tap):** This is a safe procedure that is standard care for TB meningitis. It involves taking a small sample of cerebral spinal fluid (CSF), which is the fluid that surrounds the brain and spinal cord, for examination. A sample of up to 3 teaspoons of CSF, depending upon your age and size, will be taken through a special needle in the lower part of your back. The CSF sample will be sent to a laboratory to look for the germs that cause TB meningitis and to check that the TB medicines will kill the germs.
- **Chest X-ray:** We will take an X-ray of your chest as TB can also affect the lungs and this may be seen on X-ray.
- **Sputum test:** You may be asked to cough up sputum. You may be given a salty water spray through a mask to help you cough up sputum, if required. This is a commonly used method and is safe. The sputum will be tested in a laboratory to see if it shows TB germs.
- **Blood tests:** We will take a small amount of blood, up to 4 teaspoons, depending on your age and size. The total amount of blood that we will take will never exceed a safe limit that has been agreed by the authorities. We will use some of this blood to check for other germs that could be causing your illness, that the dose of medicine given is correct and to make sure that there are no problems caused by taking the medicines (like the liver or kidneys not working well). Your HIV status will also be checked, if it is unknown, as this could mean that extra HIV treatment is needed. Study staff will tell you the results of the HIV test. We will ensure you get HIV treatment if the test is positive.
- **Urine sample:** We will store a small sample of urine (about a teaspoon) to use on a new urine test for TB.

- **Neurocognitive tests (tests to check your development):** We will use questionnaires to see if your development (e.g. walking, talking, playing and learning) was normal before the illness and to see if it has been affected by the illness. Some participants may be asked to complete some play based tasks to check their development more thoroughly.
- **Brain scan:** We will take take pictures of your brain at the start of treatment and 24 weeks later to see how the brain images change or improve with treatment, this is one of the usual investigations for anyone with tuberculosis infection of the brain. If you do not wish to take part in the study you can decline to do so by not checking the checkbox on the consent form.]

i. Getting started

If the results show that you can take the TB meningitis medicines, and you have agreed that you can join the SURE study, you will start either the standard of care treatment (12 months of medicines) or the test treatment (6 months of medicines). You will usually stay in hospital for at least 7 to 14 days or until you are well enough to eat and drink and take medicines by mouth.

j. What happens after I leave hospital?

Once you are well enough to go home you will be given enough medicine to take every day until you come back to the clinic for the planned study visits. The doctor and nurses will tell you all about these medicines and how to take them.

Someone from the SURE study team may visit you at home so that they can help remind you about planned study appointments and make sure that all is well if you are unable to come for a study appointment as planned. If they cannot visit you at home they will remain in contact by telephone.

After going home from hospital, you will need to come back regularly; 2, 4 and 8 weeks after starting treatment, then roughly every 3 months. These visits are so we can make sure you are OK and we can give you more medicines.

We know that it can be difficult taking medicines every day. We want to know how well children and adults take their medicines and if there are any problems taking them. From time to time during the study we will ask you some questions about your experiences of taking these medicines. Depending on which group you are in, after 6 or 12 months you will stop taking the TB meningitis medicines, but will continue to come to the hospital for check-ups every 3 months until 18 months from the time you started the TB meningitis treatment.

We will reimburse costs for transport to get you to the study clinic and back to your home.

k. Taking TB Meningitis medicines regularly

It is very important that:

- you do not miss any doses of the TB meningitis medicines
- the medicines are not shared with anyone else

If you do not take the TB meningitis medicines as the study team tells you, they may not work properly.

14.What are the possible side effects?

The medicines being used in the SURE study are routinely used for treating adults and children in your country for TB meningitis. Although these medicines are generally safe, side effects may occur. The most common side effects caused by the TB meningitis medicines are orange tears and urine. This is not harmful at all.

Less commonly, the following side effects may occur:

- Joint pains
- Yellow skin and eyes
- Itching skin and rash
- Vomiting or diarrhoea
- Problems with vision (very rare)
- Both aspirin and steroid medicine may cause stomach ache.

Please tell your nurse or doctor as soon as possible if you are worried about any side effects. It may be necessary to stop the medicine until the problem goes away or it is safe to re-start the medicine. We will look for side effects carefully and replace any TB meningitis medicines that cause problems.

Taking Blood: There are very few risks from taking blood samples but side effects may include discomfort, bruising and (rarely) infection. The study staff will make sure that the amount of blood taken is safe for you.

Lumbar Puncture: The risks of lumbar puncture are also few but it might be uncomfortable when the needle is inserted into your back, the possibility of infection and headache. We will ask you to lie down for a few hours after the test and make sure that you get enough fluids.

15.What are the possible benefits and disadvantages of taking part?

e. What are the possible benefits of taking part in this study?

We cannot promise that participating in the study will directly help you apart from being seen regularly by a doctor while you are in the study and being referred for further care, if needed.

However, you may benefit from taking treatment for a shorter length of time and you may also have a better recovery from TB meningitis if you are taking aspirin.

The information we get from this study will also help us to improve future treatments for children with TB meningitis.

f. What are the possible disadvantages and risks of taking part in this study?

You need to be treated for TB meningitis regardless of whether or not you join the SURE study. You might experience side effects from the medicines that you take in this study. Some of these could happen if you took these medicines even if you were not in the SURE study. Possible side effects of the TB medicines being used in the SURE study are listed above in section 5.
You will need to attend for more clinic visits than you would do if you were treated for TB meningitis outside the SURE study. You will also need to have extra blood tests.

It is important that you understand that if you join the SURE study, you will be in a research study which aims to find out whether taking the 6 month medicines works as well as the standard 1 year medicines. The medicines used in this research study are not new, are all recommended by experts and all are in use to treat TB. However, they may not all be the same as those currently used in your country's national TB treatment programme.

g. What about risks to pregnancy?

For girls who are 12 years old or above, or who have started their monthly periods a pregnancy test will be carried out before entering the study and at regular intervals during the study.

Girls should avoid getting pregnant while in the study and use effective contraceptives if you are sexually active. Some of the study drugs will reduce the effectiveness of hormonal birth control and so barrier methods of contraception (e.g. condoms) should be used.

If you do get pregnant once you have joined the study, you will be able to stay in the SURE study and receive the TB meningitis medicines. Once the baby is delivered, they will be assessed by a doctor and it maybe necessary to collect some health information about the baby.

h. What if you are living with HIV?

You can still join the SURE study if you are living with HIV.

16.Storage of blood and other samples

At some of the visits to the SURE clinic we may want to store some of the blood that was drawn so that special tests can be done later. This means that we may not give you the results of these tests at the time of your illness. We may also wish to store samples of your spinal fluid and urine for future tests.

New and better tests to diagnose TB and TB meningitis are being developed and we are asking your permission to use your samples to see how well these new tests work compared to existing ones. This includes tests to look at how your genes determine whether they are more likely to develop TB meningitis than another person and why the TB medicines work more effectively in some people than others. Genes are found in every part of the body, and passed on from mother and father to their children. They control the way we look as well as the way the body responds to different illnesses.

These genetic and other tests might be done at some point in the future and may be done in or outside your country. The tests will be decided by a group of experts. The samples taken from you will never be identified by your name and only approved people working on the study will have access to them.

You will not be given the results of findings from this genetic testing. However, any overall findings related to the presence of new genetic changes that may affect TB meningitis disease or treatment will be made available to the scientific, clinical and local community in order to improve the care and treatment of TB meningitis patients in future.

Your study nurse or doctor will be able to give you more information about this.

17.More information about taking part

d. Do I have to agree that I will take part in the SURE study?

No, it is up to you to decide whether or not you take part. If you decide to take part we will ask you to sign a consent form.

You can stop taking part in the study at any time and you won't have to give a reason.This will not affect the care you receive in any way.

b. Who is organising the study?

UCL is the sponsor for this study is organised by the Medical Research Council Clinical Trials Unit at UCL (MRC CTU), which is based in the UK and which has run studies of this kind for many years. MRC CTU will manage the study and will collect and analyse the information.

Research institutions in India, Vietnam, Uganda, Zambia and Zimbabwe are conducting the SURE study.

c. Who has reviewed the SURE study?

The study has been reviewed by international TB meningitis experts as well as by *[insert name of local Research Ethics Committee].*

d. What will happen to information about me collected during the study?

If you take part in the SURE study, the doctors and nurses will collect information whenever they see you. University College London (UCL) will be using information from you and your medical records in order to undertake this study and will act as data controller for this study. The information will be put into a computer and will be analysed by SURE researchers. The people who analyse the information will not be able to identify you and will not be able to find out your name or contact details.

Your doctor and nurses will keep your name and contact details confidential and will not pass this information to anyone outside of the clinic. They will use this information as needed, to contact you about the research study, to make sure that relevant information about the study is recorded for your care, and to oversee the quality of the study.

UCL is responsible for looking after your information and using it properly. We will follow all the laws as required to make sure that all information, including your identity and any personal details, will be kept confidential. Some of your medical information might be looked at by other authorised independent people to ensure that the SURE study is being properly carried out, but we won't share information with others that can identify you.

If the results of this study are reported or published your name will not be used nor any information that could be used to identify you. The study will be conducted in agreement with the country specific data protection regulations.

UCL is the sponsor for this study, based in the United Kingdom. University College London (UCL) will be using information from you and your medical records in order to undertake this study and will act as data controller for this study. University College London (UCL) will be responsible for looking after your information and using it properly and will keep identifiable information about you for 25 years after the study has finished.

Your hospital will collect information from you and your medical records for this research study in accordance with our instructions.

Your hospital will use your name, and contact details to contact you about the research study, and make sure that relevant information about the study is recorded for your care, and to oversee the quality of the study. UCL will collect information about you for this research study from your hospital. This information will include health information, which is regarded as a special category of information. We will use this information to conduct our research. Individuals from UCL and regulatory organisations may look at your medical and research records to check the accuracy of the research study.

For more information about UCL privacy notice, please visit:
https://www.ucl.ac.uk/legal-services/privacy/participants-health-and-care-research-privacy-notice

e. What will happen to the results of the SURE study?

We will publish a summary of the results in a medical journal. You can ask the study staff for a copy of any publication.

Results will also be discussed in community meetings and with TB meningitis and HIV programme personnel.

f. What if new information becomes available during the course of the study?

Sometimes during a study new information becomes available about the medicines that are being used. An independent committee will look at any new information and will also look at the information collected during the study and decide if any changes are needed. If this happens, your doctor will tell you about it and discuss whether you want to continue in the study. If you decide to stop taking part in the study your doctor will arrange for your care and TB treatment to continue.

g. What if something goes wrong?

If you have any concerns about the way you have been approached or treated during the SURE study, or wish to complain, please talk to the study doctors or nurses.

We do not expect that you will be injured as a result of taking part in this study. However, in the unlikely event of injury resulting from your participation in this study please contact the study coordinator on the following telephone number: *[insert number]*

The *[insert name of local IRB]* oversees the conduct of this research study which is governed by the *[insert ethical guidelines governing the study].* If you have questions about your rights as a volunteer, you can also contact the *[insert name of local IRB]* through its chairman *[insert name]* on telephone number *[insert number].*

h. What about future research?

When you agree to take part in a research study, the information about your health and care may be provided to researchers running other research studies in this organisation and in other organisations. These organisations may be universities, or companies involved in health and care research in this country or abroad. Your information will only be used by organisations and researchers to conduct research in accordance with relevant legislation, ethics and research policy requirements.

We won't share information with others that can identify you. The information will only be used for the purpose of health and care research, and cannot be used to contact you or to affect your care. It will not be used to make decisions about future services available to you, such as insurance. If there is a risk that you can be identified your data will only be used in research that has been independently reviewed by an ethics committee.

18.Contacts for further information

If you want further information about the study, contact your study doctor, nurse or counsellor (see below).

[Insert address and telephone number of study doctor and/or nurse]

Contact:_________________________

Tel:______________________________

Thank you for taking the time to consider taking part in the SURE study.

PARTICIPANT - CONSENT TO SURE STUDY

(To be printed on local-headed paper)
(Insert version no and date)

Study number:

SURE**: Short intensified treatment for children with tuberculous meningitis**

Please initial (or mark) each box if you agree

12. I confirm that I have read (or someone has read and explained to me) the information sheet *(insert version no and date)* for the SURE study and that I understand what will be required of me if I participate in the study. The study has been explained to me and my questions have been answered.	
13. I understand the SURE study will involve being seen regularly by the doctor who will ask questions about my health, and having some samples taken for testing, including an HIV test, a chest X-ray, lumbar puncture and other routine TB meningitis tests as clinically indicated.	
14. I understand that I will be given TB meningitis treatment at the recommended doses for 6 months or 12 months while I participate in the SURE study.	
15. I understand that I will be given aspirin or placebo (a pill that contains no medicine) for the first 2 months of their treatment.	
16. I understand that my participation in all aspects of this study is voluntary and that I am free to withdraw from the study at any time, without giving any reason and without my medical care or legal rights being affected.	
17. I understand that sections of any of my medical notes may be looked at by responsible individuals involved in the running of the SURE study or from regulatory authorities where it is relevant to my participation in this research. I give permission for these individuals to have access to my records, but understand that strict confidentiality will be maintained.	
18. I agree to allow samples to be taken to be stored for later testing, including genetics testing. I understand that these samples will not be identified by my name and that I will not be given results of findings from these tests relating directly to my tests, but that the overall results may be used to improve the care and treatment of TB meningitis patients in future.	
19. I agree to have brain images taken (a MRI scan) and the data being stored and used as part of the SURE study.	
20. I agree to allow the SURE care team to try and obtain information about my wellbeing from their clinic or hospital notes and the TB register at the end of the study (when the last participant enrolled has been in the study for 18 months).	
21. I agree that all information collected on me during the trial can be made available to others in the future (open access) provided no one can identify me from the details provided.	

22. I agree to taking part in the SURE study which is assessing whether it is safe and effective to have 6 months of TB meningitis treatment compared to the standard of 12 months of treatment.	

If any answers are **'no'** or you don't want to take part, **don't sign your name**.
If you **do** want to take part, you can sign your name below.

Participant's signature (or thumbprint)	Print name	Date
Witness' signature (if thumbprint used above)	Print name	Date

The person who explained the study to you needs to sign too:

I have provided the SURE study information to the participant in full and answered all his/her questions. To the best of my knowledge, he/she understands the purpose, interventions, risks and benefits of this study and willingly agrees to participate in the SURE study.		
Signature of person conducting the informed consent process	Print name	Date

IMPORTANT: one signed original to be kept in SURE trial file by the researcher
one signed copy to be given to the participant
one signed copy to be kept in the clinic file

PARTICIPANT INFORMATION SHEET AND CONSENT FORM FOR SURE + DP SUB STUDY

[To be presented on local headed paper]
[Insert: Date & Version]

SURE+DP: improving **D**iagnosis and **P**rognosis for paediatric tuberculous meningitis through the
SURE treatment trial **(ISRCTN40829906)**

Local Investigator: Dr. ……………………………………

We are inviting your child to take part in a research study

- Before you decide whether you would like your child to take part, it is important for you to understand why the research is being done and what it will involve.
- Please take time to read the following information carefully and ask the study doctor or nurse if there is anything you do not understand or you would like more details about.
- You are free to decide if you want your child to take part in this research study. If you decide that you do not want your child to take part it will not affect the care he/she receives. If your child joins the study you can later decide to withdraw him/her from the study at any time without giving a reason.
- If your child takes part in this study they will be seen by the study team while they are in hospital. We will see your child at the clinic four weeks after your child has been sent home (discharged) from the hospital to see how he/she is recovering. We will refund your transport costs.
- Thank you for taking time to read this information. If you decide you would like your child to take part we will ask you to complete a consent form and you will be given a copy of this information to keep.

Contents

10. Why are we doing this study?
11. Why have I been asked if my child can take part in the SURE+DP study?
12. What will happen if my child takes part in the SURE+DP study?
13. What are the possible side effects?
14. What are the possible benefits and disadvantages of taking part?
15. Storage of blood and other samples
16. More information about taking part
17. Contacts for further information

10.Why are we doing this study?

We are doing this study to improve the diagnosis and treatment of meningitis in children, including being able to distinguish between the different causes early on.

d. What is meningitis?

Meningitis is a serious infection of the brain, the main causes of which are infections by 'germs' especially viruses and bacteria, including tuberculosis. In children these germs can spread and damage the brain causing serious illness, severe disability and even death. Therefore, identifying the cause early and treating with the correct medicine is important to ensure the best recovery. Meningitis can be difficult to diagnose and treat effectively because it is hard to tell the different causes apart even though they need different types of treatment. In addition, some of the diagnostic tests which identify the cause are not available in many countries.

What are we trying to find out?

SURE+DP study is part of the SURE trial, a larger study that is recruiting children with meningitis caused by tuberculosis (TBM) to find a shorter and more effective treatment.
Tuberculosis is a bacterial infection which is difficult to treat and usually requires treatment for one year with many drugs. We want to find out which other infections might look similar to tuberculous meningitis and how to use different and new tests to correctly identify the different causes in a faster and easier way so that the right treatments can be given early on.

11. Why have I been asked if my child can take part in the SURE+DP study?

Your child is being invited to join this study because the doctor suspects that he/she might have meningitis.

12. What will happen if my child takes part in the SURE+DP study?

f. Screening

If the study doctor thinks that your child might have meningitis you will be asked to give your consent for your child to take part in the SURE+DP study. Your child will be examined and routine tests completed to help make the diagnosis. These tests are described below in **section 3c**.

The results of most of these tests will be ready on the same day and help the study doctor decide if your child has meningitis and the most likely cause (viral, bacterial or tuberculous) and also how sick your child is. The study doctor or nurse will discuss the results of these tests with you. Some of the special new tests will take longer to come back.

If we find that your child is sick from any other illnesses or infections (at the beginning of the study and during the study) we will make sure that your child receives the right treatment.

g. Who decides what treatments my child will get?

The treatment that your child will receive will be decided by the doctor in charge of your child and it will be the same whether you decide to participate in this study or not.

h. What tests will my child have?

Most of these tests are done for any child with suspected meningitis and are considered routine good practice.

- We will weigh your child, measure his/her height or length and the size of their head.

- **Lumbar puncture (spinal tap):** This is a common and safe procedure that is standard care for all children with meningitis. It involves taking a small sample of cerebral spinal fluid (CSF), which is the fluid that surrounds the brain and spinal cord. A sample of up to 3 teaspoons of CSF, depending upon the age and size of your child, will be taken through a special needle in the lower part of your child's back. The CSF sample will be sent to a laboratory to look for the germs that cause meningitis and, in case of bacterial infection, help the doctor choose the best medicines to kill the germs.
- **Chest X-ray:** We may take an X-ray picture of your child's lungs because some germs can also affect the lungs and these may be seen on X-ray.
- **Blood tests:** We will take a small amount of blood, up to 4 teaspoons, depending on the age and size of your child. The total amount of blood that we will take will never exceed a safe limit. We will use some of this blood to check for germs that could be causing your child's illness. Your child's HIV status will also be checked if it is unknown, as this could mean that extra HIV treatment is needed. Study staff will tell you the results of the HIV test. We will ensure your child gets HIV treatment if the test is positive.
- **Urine sample:** We will store a small sample of urine (about a teaspoon) to evaluate a urine test for TB.
- **Neurocognitive tests (tests to check your child's development):** We will use questionnaires to see if your child's development (e.g. walking, talking, playing and learning) was normal before the illness and to see if it has been affected by the illness.

i. What happens after my child is discharged from hospital?

Once your child is well enough to go home, you will follow the recommendation of your doctors and we will ask you to come back to the clinic for a study visit to see how well your child is recovering after approximately four weeks.

Someone from the SURE+DP study team will remain in contact by telephone so that they can help remind you about the planned study appointment and make sure that all is well if you are unable to bring your child to the clinic as planned. In some cases, we may visit you and your child at home.

13. What are the possible side effects?

Blood collection: There are very few risks from collecting blood but side effects may include discomfort, bruising and (very rarely) infection. The study staff will make sure that the amount of blood taken is safe for your child.

Lumbar Puncture: The risks of lumbar puncture are also few and include discomfort when the needle is inserted into the back, the possibility of infection and headache.

14. What are the possible benefits and disadvantages of taking part?

e. What are the possible benefits of taking part in this study?

There may not be a direct benefit to your child from taking part in the SURE+DP study apart from being seen by a study doctor while they are in the study. However, the information we get from this study will help us improve future diagnosis and treatment for other children with meningitis.

f. What are the possible disadvantages and risks of taking part in this study?

Your child needs to have tests sent for the causes of meningitis and other diseases regardless of whether or not they join the SURE+DP study. He/she will need to have additional samples taken at the time of standard blood tests and lumbar puncture that are routine clinical care.

g. What if my child has HIV?

Your child can still join the SURE+DP study if he or she has HIV.

15. Storage of blood and other samples

We would like to store some samples of your child's CSF, blood and urine for future tests. This means that we will not be able to give you the results of these tests at the time of your child's illness.

New and better tests to diagnose meningitis and TB meningitis are being developed and we are asking your permission to use your child's samples to see how well these new tests work to differentiate the different causes of meningitis. The tests done will be approved by a group of experts. The samples taken from your child will never be identified by their name and only approved people working on the study will have access to them. These tests may be carried out on your child’s samples in other countries with international partners.

You will not be given the results of findings from these research tests. However, any overall findings that may improve our understanding of the causes of meningitis will be made available to the scientific, clinical and local community in order to improve the care and treatment of meningitis patients in future.

Your study nurse or doctor will be able to give you more information about this.

16. More information about taking part

i. Do I have to agree that my child can take part in the SURE+DP study?

No, it is up to you to decide whether or not your child can take part. If you decide to allow your child to take part we will ask you to sign a consent form.

Your child can stop taking part in the study at any time. You won’t have to give a reason. You can also decide that your child won’t take part. This will not affect the care your child receives for their illness.

j. Who is organising the study?

This study is organised by the Medical Research Council Clinical Trials Unit at UCL (MRC CTU) which is based in the UK and which has run studies of this kind for many years. MRC CTU will manage the study and will collect and analyse the information and samples and share it with The London School of Hygiene and Tropical Medicine (LSHTM) in the UK and other research partners to conduct the research tests and analyse the data.

k. Who has reviewed the SURE+DP study?

The study has been reviewed by international meningitis experts as well as by *[insert name of local Research Ethics Committee].*

l. What will happen to information about my child collected during the study?

If your child takes part in the SURE+DP study, the doctors and nurses will collect information in accordance with our instructions whenever they see your child. The information will be put into a computer and sent to MRC CTU and LSHTM and will be analysed by SURE+DP researchers. MRC CTU, LSHTM, and any other research partners are responsible for looking after your child's information and using it properly.

We will follow all the laws and country specific data protection regulations as required to make sure that all information, including your child's identity and any personal details, will be kept confidential. No named information about you or your child will be published in any report of this study.

Your child's clinic notes may be looked at by study staff from UCL, LSHTM, relevant international regulatory authorities, the trials units co-ordinating the study or other authorised independent individuals to make sure that the SURE+DP study is being properly carried out. Confidentiality will be maintained at all times.

We won't share information with others that can identify your child. The information will only be used for the purpose of health and care research and cannot be used to contact you or to affect your child's care. It will not be used to make decisions about future services available to your child.

m. What will happen to the results of the SURE+DP study?

We will publish a summary of the results in a medical journal. You can ask the study staff for a copy of any publication.

Results will also be discussed in community meetings and with meningitis, TB and HIV programme personnel.

n. What if something goes wrong?

If you have any concerns about the way you or your child has been approached or treated during the SURE+DP study, or wish to complain, please talk to the study doctors or nurses.

We do not expect that your child will be injured as a result of taking part in this study. However, in the unlikely event of injury resulting from your child's participation in this study please contact the study coordinator on the following telephone number: *[insert number]*

The *[insert name of local IRB]* oversees the conduct of this research study which is governed by the *[insert ethical guidelines governing the study].* If you have questions about your rights or your child's rights as a volunteer, you can also contact the *[insert name of local IRB]* through its chairman *[insert name]* on telephone number *[insert number].*

Delete below if not applicable to your site
[Name of site] also has a Community Advisory Board (CAB) that helps study doctors, people in studies, and the communities where research is done to work together to look at important research issues and problems. The CAB is made up of people from the community, religious leaders, people who have been in studies and other key people. They can also help you with problems or concerns that you may have.

17.Contacts for further information

If you want further information about the study, contact your study doctor, nurse or counsellor (see below).

[Insert address and telephone number of study doctor and/or nurse]

Contact:_________________________

Tel:_____________________________

Thank you for taking the time to consider taking part in the SURE+DP study.

PARENT/CARER - CONSENT TO SURE SURE + DP SUB STUDY

(To be presented on local-headed paper)
(Insert version no and date)
Informed Consent Form - Parents and Carers

Study number:

SURE+DP: improving **D**iagnosis and **P**rognosis for paediatric tuberculous meningitis through the SURE treatment trial

Please initial (or mark) each box if you agree

13. I confirm that I have read (or someone has read and explained to me) the information sheet *(insert version no and date)* for the SURE+DP study and that I understand what will be required if my child participates in the study. The study has been explained to me and my questions have been answered.	
14. I understand the SURE+DP study will involve my child being seen by the Doctor who will ask questions about my child's health, and my child having some samples taken for testing, including an HIV test, a chest X-ray, lumbar puncture and other routine meningitis tests as clinically indicated.	
15. I understand that my child's participation in all aspects of this study is voluntary and that I am free to withdraw my child from the study at any time, without giving any reason and without his/her medical care or legal rights being affected.	
16. I understand that sections of any of my child's medical notes may be looked at by responsible individuals involved in running the SURE+DP study or from regulatory authorities where it is relevant to my child's participation in this research. I give permission for these individuals to have access to my child's records but understand that strict confidentiality will be maintained.	
17. I understand that if I am unable to continue to be the main carer for my child that I need to provide the SURE+DP study with the name of the person who will become the main carer so their consent can be requested.	
18. I agree to allow samples to be taken from my child to be stored for later testing and that this may take place in other countries. I understand that these samples will not be identified by either my or my child's name and that I will not be given results of findings from these tests relating directly to my child, but that the overall results may be used to improve the care of meningitis patients in future.	
19. I agree that all information collected on my child during the trial can be made available to others in the future (open access) provided no one can identify my child from the details provided.	
20. I agree to my child taking part in the SURE+DP study which is studying ways to improve the diagnosis of meningitis.	

Parent/carer's signature (or thumbprint)	Print name	Date

Witness's signature (if thumbprint above)	Print name	Date

I have provided the SURE+DP study information to the participant's parent/legal guardian in full and answered all his/her questions. To the best of my knowledge, he/she understands the purpose, interventions, risks and benefits of this study and willingly agrees for his/her child to be enrolled into the SURE+DP study.		
Signature of person conducting the informed consent process	Print name	Date

IMPORTANT: one signed original to be kept in SURE+DP investigator site file by the researcher
one signed copy to be given to the patient
one signed copy to be kept in the participant's clinic file

PARTICIPANT INFORMATION SHEET AND ASSENT FORM FOR CHILDREN ABLE TO GIVE ASSENT FOR SURE + DP SUB STUDY

[To be presented on local headed paper]

[Insert: Date & Version]

SURE+DP: improving **D**iagnosis and **P**rognosis for paediatric tuberculous meningitis through the
SURE treatment trial **(ISRCTN40829906)**

Local Investigator: Dr. ……………………………………

We are inviting you to take part in a research study

- Before you decide whether to take part, it is important for you to understand why we want to carry out this study and what it will mean for you.
- Please take time to read the following information carefully and ask the study doctor or nurse if there is anything you do not understand or you would like to know more.
- You are free to decide if you want to take part in this research study. If you choose not to take part it will not affect the care you receive.
- If you join the study you can stop taking part at any time without giving a reason.
- Thank you for reading this information. This copy is for you to keep.

Contents

19. Why are we doing this study?
20. Why have I been asked to take part in the SURE+DP study?
21. What will happen if I take part in the SURE+DP study?
22. What are the possible side effects?
23. What are the possible benefits and disadvantages of taking part?
24. Storage of blood and other samples
25. More information about taking part
26. Contacts for further information

19.Why are we doing this study?

We are doing this study to improve the diagnosis of meningitis.

g. What is meningitis?

Meningitis is a serious infection of the brain caused by viruses and bacteria. The infection can be difficult to differentiate from other diseases that affect the brain and, as a result of the delay in treatment, children may get severely ill.

e. What are we trying to find out?

The diagnosis of meningitis is difficult because the presentation is similar to other infectious and non-infectious diseases which affect the brain. We want to find out how to use new tests to differentiate the different causes of meningitis more quickly and easily.

20.Why have I been asked to take part in the SURE+DP study?

You have been invited to join this study because your doctor considers you might have meningitis.

21. What will happen if you take part in the SURE+DP study?

l. Screening

If the study doctor suspects that you might have meningitis, you will be asked to sign an assent form to show that you agree to take part in the SURE+DP study. Your parent/guardian will be asked to sign a consent form to show you have both understood the information given.

You will be examined by a doctor who will do some further tests to evaluate the cause of your illness. These tests are described below in section 3b.

The results of most of these tests will be ready on the same day and will help the doctor find out if you have meningitis.

If we find that you are sick from any other illnesses or infections (at the beginning of the study and during the study) your doctor will make sure that you receive the right treatment.

m. What tests will I have?

Most of these tests are done for any child or young person with suspected meningitis and are considered good practice.

- We will weigh you and measure your height and the size of your head.
- **Lumbar puncture:** This procedure involves taking a small sample of the fluid that surrounds the brain and spinal cord through a special needle in your back. We will take up to 3 teaspoons (15mls) of fluid, depending upon your age and size. The sample will be sent to a laboratory to look for the germs that cause meningitis.
- **Chest X-ray:** We might take an X-ray picture of your chest because some germs can also affect the lungs, which may be seen on X-ray.
- **Blood tests:** We will take a small amount of blood, up to 3 teaspoons (15mls), depending upon your age and size. The total amount of blood taken will never exceed a safe limit. We will use some of this blood to check for other germs that could be causing your illness.
- **Urine sample:** We will collect and store a small sample of urine (about a teaspoon) to use on a urine test for TB.
- **Neurocognitive tests (tests to check your development):** We will use questionnaires to see if your development (e.g. walking, talking, playing and learning) was OK before the illness and to see if it has been affected by the illness.

n. What happens after I leave hospital?

Once you are well enough to go home you will follow the recommendation of your doctors and we will ask that you come back to the clinic for a study visit so we can make sure you are OK.

22. What are the possible side effects?

Taking Blood: There are very few risks from taking blood samples but side effects may include discomfort, bruising and (rarely) infection. The study staff will make sure that the amount of blood taken is safe for you.

Lumbar Puncture: The risks of lumbar puncture are also few but it might be uncomfortable when the needle is inserted into your back. We will ask you to lie down for a few hours after the test and make sure that you get enough fluids.

23.What are the possible benefits and disadvantages of taking part?

i. What are the possible benefits of taking part in this study?

We cannot promise that participating in the study will directly help you apart from being seen by a study doctor while you are in the study and being referred for further care, if needed.

The information we get from this study will also help us to improve future diagnosis for children with meningitis.

j. What are the possible disadvantages and risks of taking part in this study?

You need to have tests for meningitis and other diseases regardless of whether or not you join the SURE+DP study. Additional samples will be taken at the same time as these tests.

k. What if you are living with HIV?

You can still join the SURE+DP study if you are living with HIV.

24.Storage of blood and other samples

We may want to store some of the blood that was drawn so that special tests can be done later. We may also wish to store samples of your spinal fluid and urine for future tests. Some of these tests might be carried out in other countries.

Your study nurse or doctor will be able to give you more information about this.

25.More information about taking part

e. Do I have to agree that I will take part in the SURE+DP study?

No, it is up to you to decide whether or not you take part. This will not affect the care you receive in any way.

You can stop taking part in the study at any time and you won't have to give a reason.

f. What will happen to information about me collected during the study?

If you take part in the SURE+DP study, the doctors and nurses will collect information about you. The information will be put into a computer and will be analysed by SURE+DP researchers. The people who analyse the information will not be able to identify you and will not be able to find out your name or contact details.

Your doctor and nurses will keep your name and contact details confidential and will not pass this information to anyone outside of the clinic. They will use this information as needed, to contact you about the research study, to make sure that relevant information about the study is recorded for your care, and to oversee the quality of the study.

Medical Research Council Clinical Trials Unit at UCL (MRC CTU) and the London School of Hygiene and Tropical Medicine (LSHTM), which are based in the UK and other approved research are responsible for looking after your information and using it properly. We will follow all the laws as required to make sure that all information, including your identity and any personal details, will be kept confidential. Some of your medical information might be looked at by other authorised independent people to ensure that the SURE+DP study is being properly carried out, but we won't share information with others that can identify you.

If the results of this study are reported or published your name will not be used nor any information that could be used to identify you. The study will be conducted in agreement with the country specific data protection regulations.

26.Contacts for further information

If you want further information about the study, contact your study doctor, nurse or counsellor (see below).

[Insert address and telephone number of study doctor and/or nurse]

Contact:_________________________

Tel:_____________________________

Thank you for taking the time to consider taking part in the SURE+DP study.

PARENT/CARER - CONSENT TO SURE + DP SUB STUDY

(To be presented on local-headed paper)
(Insert version no and date)
Informed Consent Form - Parents and Carers

Study number:

SURE+DP: improving **D**iagnosis and **P**rognosis for paediatric tuberculous meningitis through the SURE treatment trial

Please initial (or mark) each box if you agree

1. I confirm that I have read (or someone has read and explained to me) the information sheet *(insert version no and date)* for the SURE+DP study and that I understand what will be required if my child participates in the study. The study has been explained to me and my questions have been answered.	
2. I understand the SURE+DP study will involve my child being seen by the Doctor who will ask questions about my child's health, and my child having some samples taken for testing, including an HIV test, a chest X-ray, lumbar puncture and other routine meningitis tests as clinically indicated.	
3. I understand that my child's participation in all aspects of this study is voluntary and that I am free to withdraw my child from the study at any time, without giving any reason and without his/her medical care or legal rights being affected.	
4. I understand that sections of any of my child's medical notes may be looked at by responsible individuals involved in running the SURE+DP study or from regulatory authorities where it is relevant to my child's participation in this research. I give permission for these individuals to have access to my child's records but understand that strict confidentiality will be maintained.	
5. I understand that if I am unable to continue to be the main carer for my child that I need to provide the SURE+DP study with the name of the person who will become the main carer so their consent can be requested.	
6. I agree to allow samples to be taken from my child to be stored for later testing and that this may take place in other countries. I understand that these samples will not be identified by either my or my child's name and that I will not be given results of findings from these tests relating directly to my child, but that the overall results may be used to improve the care of meningitis patients in future.	
7. I agree that all information collected on my child during the trial can be made available to others in the future (open access) provided no one can identify my child from the details provided.	
8. I agree to my child taking part in the SURE+DP study which is studying ways to improve the diagnosis of meningitis.	

Parent/carer's signature (or thumbprint)	Print name	Date

Witness's signature (if thumbprint above)	Print name	Date

I have provided the SURE+DP study information to the participant's parent/legal guardian in full and answered all his/her questions. To the best of my knowledge, he/she understands the purpose, interventions, risks and benefits of this study and willingly agrees for his/her child to be enrolled into the SURE+DP study.		
Signature of person conducting the informed consent process	Print name	Date

IMPORTANT: one signed original to be kept in SURE+DP investigator site file by the researcher
one signed copy to be given to the patient
one signed copy to be kept in the participant's clinic file

PARTICIPANT - ASSENT TO SURE+DP STUDY

(To be printed on local-headed paper)
(Insert version no and date)

Study number:

SURE+DP: improving **D**iagnosis and **P**rognosis for paediatric tuberculous meningitis through the SURE treatment trial

Please tick yes or no to the questions below

1. Have you read (or had read to you) the information about this study?	YES ☐ NO ☐
2. Do you understand what the study is about?	YES ☐ NO ☐
3. Have you asked all the questions you want?	YES ☐ NO ☐
4. Have your questions been answered in a way you understand?	YES ☐ NO ☐
5. Can we perform a lumbar puncture on you?	YES ☐ NO ☐
6. Do you understand the risks and benefits of taking part in the study?	YES ☐ NO ☐
7. Do you understand that you are free to stop the study at any time?	YES ☐ NO ☐
8. Are you happy to take part?	YES ☐ NO ☐

If any answers are **'no'** or you don't want to take part, **don't sign your name**.
If you **do** want to take part, you can sign your name below.

Participant's signature (or thumbprint)	Print name	Date
Witness' signature (if thumbprint used above)	Print name	Date

The person who explained the study to you needs to sign too:

I have provided the SURE+DP study information to the participant in full and answered all his/her questions. To the best of my knowledge, he/she understands the purpose, interventions, risks and benefits of this study and willingly agrees to participate in the SURE+DP study.

Signature of person conducting the informed consent process	Print name	Date

IMPORTANT: one signed original to be kept in SURE+DP trial file by the researcher
one signed copy to be given to the participant
one signed copy to be kept in the clinic file

PARTICPANT INFORMATION SHEET AND CONSENT FORM FOR PHARMACOKINETIC STUDY

[To be presented on local headed paper]

[Insert: Date & Version]

SURE PK Sub study

We are inviting your child to take part in an additional part of the SURE research study – a pharmacokinetic (PK) sub study.

1. Why are we doing this substudy?

This sub study is part of the SURE study, which your child is already a part of. It is measuring the amount of anti-TB meningitis medicine in the blood and CSF (fluid surrounding the brain) of children taking the treatment for 6 months.

2. Why have I been asked if my child would like to take part in this PK study?

You have been asked to give permission for your child to take part in this substudy as they are already part of the SURE study and have been allocated the 6 month TB meningitis treatment.

In total 36 children from the SURE study will be included in this substudy.

3. What will happen if we decide not to take part?

It is totally up to you and your child whether or not he/she takes part. If you or your child decides not to take part in the PK study this will not stop your child from taking part in SURE or affect your child's care now or in the future in any way.

4. What will happen if my child takes part in the SURE PK substudy?

If you decide to take part, we will go through this information sheet together. We will give you a copy to keep. You will be asked to sign a consent form to show you understand the information and agree to take part.

If your child does take part, blood samples will be taken from your child and stored so researchers can learn more about anti-TB meningitis medicines. In addition, in some children we will collect a small amount of the fluid that surrounds the brain and spinal cord, to determine the levels of TB drugs reaching the site of the TB infection. If at any subsequent point your child wants to stop being in the study we hope that you will still allow us to use these samples. If, however, you do not give your permission then we will destroy them.

Important to know

- ***If your child is eligible and does take part they will need to spend one whole day in the hospital/clinic around 2 weeks after starting treatment with the anti-TB meningitis medicines. Many children will still be receiving their treatment in hospital at this time.***

If your child is no longer in hospital they will need to be brought back in for a full day visit 2 weeks after they started their treatment. This is for the PK substudy blood and CSF sampling day.

We will reimburse your transport costs for this visit if your child is not still in hospital. An appointment will be made for your child to spend a day (approximately 12 hours) at the hospital. You will be given an appointment date and a time so you can plan the visit.

On the day booked for the blood sampling visit your child will be asked to come to the clinic for a whole day:

- He/she will have up to 7 blood samples taken during the day (over a 12 hour period) to test the level of medicine in their blood. A cream with local pain-killing effect will be used to make the skin numb and a thin flexible tube (called a cannula) will be inserted into a vein and kept in place for the day to enable the blood samples to be taken more easily. All blood samples will be taken from the cannula so only one needle prick is required. Each blood sample will be small (less than half a teaspoon), up to a total of no more than 6ml (less than 1½ teaspoons) of blood. The amount will be safe for your child's age and weight.

- We will also perform a lumbar puncture - This procedure involves taking a small sample of the fluid that surrounds the brain and spinal cord. We will take up to 3 teaspoons, depending upon your child's age and size, through a special needle placed in the lower back. The sample will be sent to the laboratory to look for the germs that cause TB meningitis and to measure the amount of TB medicines in the CSF and reaching the site of infection.

At this point the PK (blood and CSF levels) study will end. However, your child's usual SURE visits will continue for routine monitoring of their health and recovery from the TB meningitis.

It is very important that your child attends the clinic on time on the day of their PK blood draw, and that you have kept an accurate record of the time that they took their medicines on the day before the PK blood draw visit. Please also remember not to give them their medicines on the morning that they attend the PK visit – the drugs will be given at the clinic by study staff.

5. What are the possible benefits of taking part in this study?

There will not be a direct benefit to your child from taking part in this study but the results of this study will give us more information about the dosing of TB meningitis medicines for children and young people. It is very important that we have this information so we can make sure that children are given the correct amount of medicine.

This information will help children and young people across the world with TB meningitis get the most appropriate amount of medicine to treat the disease effectively.

6. What are the possible disadvantages and risks of taking part in this study?

It is important to think about these before deciding to take part.

This study will not result in any risk to your child's health. However, your child will have to spend one day (around 12 hours) at the hospital/clinic. In some cases your child will be required to stay on the ward for the night before their PK visit. You will be reimbursed for transport costs and for the inconvenience of the length of this visit.

On the PK day, up to 7 small blood samples will be taken but only one needle prick will be needed, and a lumbar puncture will be completed to collect a small sample of CSF.

7. More information about taking part

Who has checked this study is safe?

This study has been looked at by an independent group of people (a Research Ethics Committee) to make sure that, as far as possible, the safety and wellbeing of anyone taking part in the study is protected.

A second group (the Independent Data Monitoring Committee) will review the results as they come out and will recommend if the study is safe to continue. This is standard for all studies looking at medicines for adults or children.

What if something goes wrong?

If you have any concerns about the way you or your child has been approached or treated during the study, or wish to complain, please talk to the study doctors or nurses.

The [insert name of local IRB] oversees the conduct of this research study which is governed by the [insert ethical guidelines governing the study]. If you have questions about your rights or your child's rights as a volunteer, you can also contact the [insert name of local IRB] through its chairman [insert name] on telephone number [insert number].

What will happen to information about my child collected during the study?

If your child takes part in the SURE PK study, the doctors and nurses will collect information and blood and CSF samples on the day of the PK visit.
The information will be put into a computer and will be analysed by SURE researchers.
The blood and CSF samples collected as part of the PK substudy will be analysed at Radboud University in The Netherlands.

We will follow all the laws as required to make sure that all information, including your child's identity and any personal details, will be kept confidential. No named information about you or your child will be published in any report of this study.

8. Contacts for further information

If you want further information about the study, contact your study doctor, nurse or counsellor (see below).
[Insert address and telephone number of study doctor and/or nurse]

Contact: ___________________________
Tel: _______________________________

Thank you for taking the time to consider taking part in this PK sub study.

PARENT/CARER - CONSENT TO SURE PHARMACOKINETIC (PK) STUDY

Study number:

(To be printed on local-headed paper)
(Insert version no and date)

SURE: Short intensified treatment for children with tuberculous meningitis

Please initial (or mark) box if you agree:

I have read/been read the information sheet (version ____, dated __________) for the SURE PK (blood levels) study and I understand what will be required if my child participates in the study.	
The PK study has been explained to me and my questions have been answered.	
I understand that my child's participation in this study is voluntary and that I am free to withdraw him or her at any time, without giving any reason, without my medical care or legal rights or my child's medical care or legal rights being affected.	
I agree for small quantities of blood and a CSF sample to be stored with anonymised labelling so that further work can be done to help understand more about TB meningitis treatment. *[Country specific, delete if not applicable]*	
I agree to study data, including my child's study number and age, being transferred to xxx, and to drug agencies in other countries.	
I agree for my child to participate in the SURE PK (blood levels) study if they are eligible.	

Parent/carer's signature (or thumbprint)	Print name	Date
Witness' signature (if thumbprint above)	Print name	Date

I have provided the SURE PK study information to the participant's parent/legal guardian in full and answered all his/her questions. To the best of my knowledge, he/she understands the purpose, interventions, risks and benefits of this study and willingly agrees for his/her child to participate in the SURE PK study.		
Signature of person conducting the informed consent process	Print name	Date

IMPORTANT: one signed original to be kept in SURE trial file by the researcher
one signed copy to be given to the parent/carer
one signed copy to be kept in the clinic file
