# Supplementary material for: Effectiveness and safety of shortened intensive treatment for children with tuberculous meningitis (SURE): a protocol for a phase 3 randomised controlled trial evaluating 6 months of antituberculosis therapy and 8 weeks of aspirin in Asian and African children with tuberculous meningitis
Source: BMJ Open. 2025 Apr 2;15(4):e088543. doi: 10.1136/bmjopen-2024-088543 (PMC11967003; doi:10.1136/bmjopen-2024-088543)
Supplement: online supplemental file 2 [file bmjopen-15-4-s002.docx]

**Table S1a: SURE study schedule (screening, randomisation, day 0-14)**

|  |  |  | **Days following enrolment** | | | | | | | | | | | | | |
| --- | --- | --- | --- | --- | --- | --- | --- | --- | --- | --- | --- | --- | --- | --- | --- | --- |
| **All children** | **Screening** | **Randomisation** | **1** | **2** | **3** | **4** | **5** | **6** | **7** | **8** | **9** | **10** | **11** | **12** | **13** | **14** |
| Dispensing TBM drugs |  | X | (X) | (X) | (X) | (X) | (X) | (X) | (X) | (X) | (X) | (X) | (X) | (X) | (X) | X |
| Consent and patient information | X |  |  |  |  |  |  |  |  |  |  |  |  |  |  |  |
| History and physical examination | X | X | X | X | X | X | X | X | X | X | X | X | X | X | X | X |
| Clinical measurements (temperature, heart rate, respiratory rate, blood pressure, seizure frequency) | X | X | X | X | X | X | X | X | X | X | X | X | X | X | X | X |
| Anthropometry | X | X |  |  |  |  |  |  |  |  |  |  |  |  |  | X |
| Blantyre Coma Score (BCS) | X |  | X | X | X | X | X | X | X | X | X | X | X | X | X | X |
| modified Rankin Scale (mRS) |  |  |  |  |  |  |  |  | X |  |  |  |  |  |  |  |
| Liverpool outcome score (LOS) |  |  |  |  |  |  |  |  | X |  |  |  |  |  |  |  |
| BMRC TBM severity grade* | X |  |  |  |  |  |  |  | X |  |  |  |  |  |  |  |
| Lumbar Puncture | X |  |  |  |  |  |  |  | (X) |  |  |  |  |  |  |  |
| CSF chemistry (glucose and total protein) and microscopy | X |  |  |  |  |  |  |  | (X) |  |  |  |  |  |  |  |
| CSF bacterial and mycobacterial culture (and Xpert MTB/RIF if available) | X |  |  |  |  |  |  |  | (X) |  |  |  |  |  |  |  |
| Haematology (FBC) | X |  |  |  |  |  |  |  | X |  |  |  |  |  |  | X |
| Biochemistry (ALT, bilirubin, Na+, K+, urea, creatinine, glucose – paired with CSF glucose) | X |  |  |  |  |  |  |  | X |  |  |  |  |  |  | X |
| Blood for bacterial culture | X |  |  |  |  |  |  |  | (X) |  |  |  |  |  |  |  |
| Blood for HIV test unless known to be HIV positive (PCR if <18 months of age) | X |  |  |  |  |  |  |  |  |  |  |  |  |  |  |  |
| Chest X-Ray (CXR) –antero-posterior (AP) or PA if possible | X |  |  |  |  |  |  |  |  |  |  |  |  |  |  |  |
| Gastric aspirate (Gastric aspirate (EMGW) / induced sputum for microscopy, culture and DST [12] (and Xpert MTB/RIF if available) | X | X | (X) | (X) |  |  |  |  |  |  |  |  |  |  |  |  |
| Any other suspected clinical sites of TB disease investigated as appropriate | X |  |  |  |  |  |  |  |  |  |  |  |  |  |  |  |
| Urine Pregnancy Test (girls ≥12 years or who have started menses) | X |  |  |  |  |  |  |  |  |  |  |  |  |  |  |  |
| CT/MRI brain | X |  |  |  |  |  |  |  |  |  |  |  |  |  |  |  |
| **Additional tests for children living with HIV** | **Screening** | **Randomisation** | **1** | **2** | **3** | **4** | **5** | **6** | **7** | **8** | **9** | **10** | **11** | **12** | **13** | **14** |
| Viral load / plasma storage | (X) | X |  |  |  |  |  |  |  |  |  |  |  |  |  |  |
| CD4 count | (X) | X |  |  |  |  |  |  |  |  |  |  |  |  |  |  |
| CSF CRAG test (or India Ink) if CD4 count <100 cells/ µL | (X) | X |  |  |  |  |  |  |  |  |  |  |  |  |  |  |
| WHO clinical disease staging | (X) | X |  |  |  |  |  |  |  |  |  |  |  |  |  |  |
| Detail of ART regimen | (X) | X |  |  |  |  |  |  |  |  |  |  |  |  |  |  |

**Table S1b: SURE study schedule (week 4 – 72 follow up)**

|  | **Weeks from randomisation** | | | | | | |
| --- | --- | --- | --- | --- | --- | --- | --- |
| **All children** | **4** | **8** | **16** | **24** | **36** | **48** | **72** |
| Dispensing TBM drugs | X | X | X | X | X |  |  |
| History and physical examination | X | X | X | X | X | X | X |
| Clinical measurements (temperature, seizure frequency) | X | X | X | X | X | X | X |
| Anthropometry | X | X | X | X | X | X | X |
| Blantyre coma score (BCS) | X | X | X | X | X | X | X |
| modified Rankin Scale (mRS) |  |  |  | X |  | X | X |
| Liverpool Outcome Score (LOS) |  |  |  | X |  | X | X |
| Adherence and acceptability assessment |  | X |  | X |  | X |  |
| Haematology (FBC) | X | X |  |  |  |  |  |
| Biochemistry (AST or ALT, bilirubin, Na+, K+, urea, creatinine | X | X |  |  |  |  |  |
| Urine Pregnancy Test (girls ≥12 years or who have started menses) |  |  |  | X |  | X | X |
| **Additional tests for children living with HIV** | **4** | **8** | **16** | **24** | **36** | **48** | **72** |
| Viral load / plasma storage |  |  |  | X |  | X | X |
| CD4 count |  |  |  | X |  | X | X |
| WHO clinical disease staging |  |  |  | X |  | X | X |

***MRC TBM grade = medical research council grading of TBM. Grade 1 severity is defined as Glasgow Coma Scale 15 without focal neurology, Grade 2 severity as Glasgow Coma Scale 15 with focal neurology or Glasgow Coma Scale 11-14 with/without focal neurology, and Grade 3 severity as GCS of 10 or less with or without focal neurology**

**INTERVENTION ARM (6 MONTHS)**

Table S2a. Drug dosing and drug exposure for children in the intervention arm, weight bands 3.0-24.9kg (modified WHO weight bands), using paediatric dispersible FDC HR 50/75, dispersible Z 150 and L 250

| Weight bands* kg | Number of tablets | | | Target dose ** mg/kg | | | |
| --- | --- | --- | --- | --- | --- | --- | --- |
|  | HR 50/75 | Z150 | L250 | H20 | R30 | Z40 | L20 |
|  |  |  |  | Achieved Dose, mg/kg | | | |
| 3.0-3.9 | 1.5 | 0.5 | 0.25 | 19 -25 | 29-38 | 19-25 | 16-21 |
| 4.0-5.9 | 2 | 1 | 0.25 | 17-25 | 25-38 | 25-38 | 11-16 |
| 6.0-7.9 | 3 | 2 | 0.5 | 19-25 | 28-38 | 38-50 | 16-21 |
| 8.0-11.9 | 4 | 2 | 0.75 | 17-25 | 25-38 | 25-38 | 16-23 |
| 12.0-15.9 | 6 | 3 | 1 | 19-25 | 28-38 | 28-38 | 16-21 |
| 16.0-19.9 | 8 | 4 | 1.5 | 20-25 | 30-38 | 30-38 | 19-23 |
| 20.0-24.9 | 8 | 5 | 2 | 16-20 | 24-30 | 30-38 | 20-25 |

* Modified WHO weight bands adapted to achieve adequate doses;

** Target dosing range is increased compared to WHO-recommended doses. Target dosing - H: 20mg/kg, maximum dose 400mg/day; R: 30mg/kg, maximum dose 600mg/day; Z: 40mg/kg, maximum dose 750mg/day; L: 20mg/kg, maximum dose 500mg/day

Table S2b: Drug dosing and drug exposure for children in the intervention arm, weight ≥25kg (modified WHO weight bands), using adult FDC HR 75/150, Z 400 and L 250

| Weight bands*  kg | Number of tablets | | | Target dose**, mg/kg | | | |
| --- | --- | --- | --- | --- | --- | --- | --- |
|  |  |  |  | H 10 | R 20 | Z 30 | L 20 |
|  | HR  75/150 | Z  400 | L  250 | Achieved dose, mg/kg | | | |
| 25-32.9 | 6 | 2 | 2 | 14-18 | 27-36 | 24-32 | 16-20 |
| 33-54.9 | 6 | 3 | 3 | 8-14 | 16-27 | 22-36 | 14-23 |
| 55-70 | 8 | 4 | 3 | 9-11 | 17-22 | 23-29 | 11-14 |
| >70 | 8 | 5 | 4 | ≤9 | ≤17 | ≤28 | ≤14 |

* Modified WHO weight bands adapted to be consistent across both treatment arms;

**Target dosing range is increased compared to WHO-recommended doses. Target dosing - H: 10mg/kg, maximum dose 600mg/day; R: 20mg/kg, maximum dose 1200mg/day; Z: 30mg/kg, maximum dose 2000mg/day; L: 20mg/kg, maximum dose 1000mg/day

**CONTROL ARM (12 MONTHS)**

Table S3a. Drug dosing and drug exposure for children in the control arm intensive phase, weight bands 3.0-24.9kg (modified WHO weight bands), based on paediatric WHO guidelines, using paediatric FDC (HRZ 50/75/150)

| Weight bands*, kg | Number of tablets | | Target dose**, mg/kg | | | |
| --- | --- | --- | --- | --- | --- | --- |
|  |  |  | H 10 | R 15 | Z 35 | E 20 |
|  | HRZ 50/75/150 | E 100 | Achieved dose, mg/kg | | | |
| 3.0-3.9 | 0.75 | 0.75 | 10-13 | 14-19 | 29-38 | 19-25 |
| 4.0-5.9 | 1 | 1 | 8-13 | 13-19 | 25-38 | 17-25 |
| 6.0-7.9 | 1 | 1 | 6-8 | 9-13 | 19-25 | 13-17 |
| 8.0-11.9 | 2 | 2 | 8-13 | 13-19 | 25-38 | 17-25 |
| 12.0-15.9 | 3 | 3 | 9-13 | 14-19 | 28-38 | 19-25 |
| 16.0-24.9 | 4 | 4 | 8-13 | 12-19 | 24-38 | 16-25 |
| * Modified WHO weight bands adapted to be consistent across both treatment arms;  **Target dosing - H: 10mg/kg (range 7–15mg/kg) maximum dose 200mg/day; R:15mg/kg (range 10–20mg/kg) maximum dose 300mg/day; Z: 35mg/kg (30–40mg/kg) maximum dose 600mg/day; E: 20mg/kg (15-25mg/kg) maximum dose 400mg/day | | | | | | |

Table S3b. Drug dosing and drug exposure for children in the control arm intensive phase, weight ≥ 25kg (modified WHO weight bands), based on paediatric WHO guidelines using adult FDC (HRZE 75/100/400/275)

| Weight bands*, kg | Number of tablets | Target dose**, mg/kg | | | |
| --- | --- | --- | --- | --- | --- |
|  |  | H 5 | R 10 | Z 25 | E 20 |
|  | HRZE 75/150/400  /275 | Achieved dose, mg/kg | | | |
| 25.0-32.9 | 2 | 5-6 | 9-12 | 24-32 | 17-22 |
| 33-54.9 | 3 | 4-7 | 8-14 | 22-36 | 15-25 |
| 55-70 | 4 | 4-6 | 9-11 | 23-29 | 16-20 |
| >70 | 5 | ≤5 | ≤11 | ≤28 | ≤19 |
| *Modified WHO weight bands adapted to be consistent across both treatment arms;  ** Target dosing - H: 5mg/kg (range 4–6mg/kg) to a maximum dose 375mg/day; R:10mg/kg (range 8–12mg/kg) to a maximum dose 750mg/day; Z: 25mg/kg (20–30mg/kg) to a maximum dose 2000mg; E: 20mg/kg (range 15-25mg/kg) to a maximum dose 1375mg. | | | | | |

Table S3c Drug dosing and drug exposure for children in the control arm continuation phase, weight bands 3.0-24.9kg, based on paediatric WHO guidelines using new paediatric FDC (HR 50/75)*

| Weight bands*, kg | Number of tablets | Target dose**, mg/kg | |
| --- | --- | --- | --- |
|  |  | H 10 | R 15 |
|  | HR 50/75 | Achieved dose, mg/kg | |
| 3.0-3.9 | 0.75 | 10-13 | 14-19 |
| 4.0-5.9 | 1 | 8-13 | 13-19 |
| 6.0-7.9 | 1 | 6-8 | 9-13 |
| 8.0-11.9 | 2 | 8-13 | 13-19 |
| 12.0-15.9 | 3 | 9-13 | 14-19 |
| 16.0-24.9 | 4 | 8-13 | 12 -19 |
| *Modified WHO weight bands adapted to be consistent across both treatment arms;  ** Target dosing - H: 10mg/kg (range 7–15mg/kg) to a maximum dose 200mg/day; R:15mg/kg (range 10–20mg/kg) to a maximum dose 300mg/day | | | |

Table S3d. Drug dosing and drug exposure for children in the control arm continuation phase, weight ≥25kg, based on WHO adult guidelines using adult FDC (HR 75/150)*

| Weight bands*, kg | Number of tablets | Target dose**, mg/kg | |
| --- | --- | --- | --- |
|  |  | H 5 (4-6) | R 10 (8-12) |
|  | HR 75/150 | Achieved dose, mg/kg | |
| 25–32.9 | 2 | 5-6 | 9-12 |
| 33 – 54.9 | 3 | 4-7 | 8-14 |
| 55 - 70 | 4 | 4-6 | 9-11 |
| >70 | 5 | ≤5 | ≤11 |
| *Modified WHO weight bands adapted to be consistent across both treatment arms;  ** Target dosing - H: 5mg/kg (range 4–6mg/kg) to a maximum dose 375mg/day; R:10mg/kg (range 8–12mg/kg) to a maximum dose 150mg/day | | | |

Table S4. Aspirin dosing and drug exposure for children in the intervention arm using dispersible (80mg) aspirin/placebo

| **Weight bands, kg** | **Number of 80mg aspirin tablets** | **Target dose 20mg/kg** |
| --- | --- | --- |
|  |  | **Achieved dose mg/kg** |
| 3.0-3.9 | 0.5 | 10-13 |
| 4.0-7.9 | 1 | 10-20 |
| 8.0-11.9 | 2 | 13-20 |
| 12.0-15.9 | 3 | 15-20 |
| 16.0-19.9 | 4 | 16-20 |
| 20.0-24.9 | 5 | 16-20 |
| 25.0-32.9 | 6 | 15-19 |
| 33.0-54.9 | 8 | 12-19 |
| 55.0-70.0 | 10 | 11-15 |
| ≥70 | 12 | ≤14 |

**Table S5. Definitions used for safety reporting**

| **Term** | **Definition** |
| --- | --- |
| Adverse Event (AE) | Any untoward medical occurrence in a patient or clinical trial subject to whom medicinal product has been administered including occurrences that are not necessarily caused by or related to that product. |
| Adverse Reaction (AR) | Any untoward and unintended response to an investigational medicinal product related to any dose administered. |
| Unexpected Adverse Reaction (UAR) | Ad adverse reaction, the nature of severity of which is not consistent with the information about the medicinal product in question set out in the Summary of Product Characteristics (SPC) or investigator Brochure (IB). |
| Serious Adverse Event (SAE) | Respectively an adverse event, adverse reaction or unexpected adverse reaction that:   - Results in death - Is life-threatening* - Requires hospitalisation or prolongation of existing hospitalisation** - Results in persistent or significant disability or incapacity - Consists of a congenital anomaly or birth defect - Is another important medical condition*** |
| Serious Adverse Reaction (SAR) | A SAE where causality has been assessed as possible, probable or definitely related to the drug by the investigator |
| Serious Unexpected Serious Adverse Reaction (SUSAR) | A SAR where the nature or severity of which is not consistent with the information about the medicinal product in question set out in the summary of product characteristics (SPC) or investigator brochure (IB) for that product |

* life-threatening = an event in which the patient is at risk of death at the time of the event; does not refer to an event that hypothetically might cause death if it were more severe, for example, a silent myocardial infarction.

**Hospitalisation = inpatient admission, regardless of length of stay, even if the hospitalisation is a precautionary measure for continued observation. Hospitalisations for a pre-existing condition, that has not worsened or for an elective procedure do not constitute an SAE.

*** Medical judgement required. The following is also considered serious: important AEs or ARs that are not immediately life-threatening or do not result in death or hospitalisation but may jeopardise the subject or may require intervention to prevent one of the other outcomes listed in the definition above.

**Table S6 Pre-specified secondary endpoints for End-Point review**

| Gastrointestinal bleeding (any grade) |
| --- |
| Development of obstructive hydrocephalus |
| Clinical or microbiological relapse of TBM and/or TB disease at other sites by 72 weeks |
| Acquired drug resistance |
| Events of interest (EOI) that meet the above criteria including tendonitis |
| Any new grade 3 or 4 clinical or laboratory adverse events and adverse events (any grade) leading to treatment modification |
