# Supplementary material for: Effectiveness and safety of shortened intensive treatment for children with tuberculous meningitis (SURE): a protocol for a phase 3 randomised controlled trial evaluating 6 months of antituberculosis therapy and 8 weeks of aspirin in Asian and African children with tuberculous meningitis
Source: BMJ Open. 2025 Apr 2;15(4):e088543. doi: 10.1136/bmjopen-2024-088543 (PMC11967003; doi:10.1136/bmjopen-2024-088543)
Supplement: Supplementary file 3 [file bmjopen-2024-088543.reviewer_comments.pdf]

PEER REVIEW HISTORY

BMJ Open publishes all reviews undertaken for accepted manuscripts. Reviewers are asked to complete a checklist review form and are provided with free text boxes to elaborate on their assessment. These free text comments are reproduced below.

ARTICLE DETAILS

Title (Provisional)

Effectiveness and safety of shortened intensive treatment for children with tuberculous meningitis (SURE): protocol for a phase 3 randomised controlled trial evaluating 6 months of anti-tuberculosis therapy and 8 weeks of aspirin in Asian and African children with tuberculous meningitis

Authors

Huynh, Julie; Chabala, Chishala; Sharma, Suvasini; Choo, Louise; Singh, Varinder; Sankhyan, Naveen; Mujuru, Hilda; Nguyen, Nhung; Trinh, Tung; Phuc, Phan Huu; Nhung, Nguyen Viet; Nkole, Kafula Lisa; Sirari, Titiksha; Mutata, Constantine; Frangou, Elena; Griffiths, Anna; Wobudeya, Eric; Muller, Caitlin; Santana, Sierra; Kestelyn, Evelyne; Nguyen, Lam; Nguyen, Thanh; Tran, Dai; Seddon, James; Turkova, Anna; Abarca-Salazar, Susan; Basu-Roy, Robin; Thwaites, Guy E; Crook, Angela; Anderson, Suzanne; Gibb, Diana; SURE, trial team

---

VERSION 1 - REVIEW

**Reviewer**	**1**
**Name**	**Schaaf, Simon**
**Affiliation**	**Desmond Tutu TB Centre, Stellenbosch University, Paediatrics and Child Health**
**Date**	**09-Jul-2024**
**COI**	**No competing interest**

Review MS: SHORTENED INTENSIVE TREATMENT FOR CHILDREN WITH TUBERCULOUS MENINGITIS (SURE): a study protocol

The study is an important one to lead the way for shorter treatment for tuberculous meningitis in children with medicines that are readily available in low- and middle-income countries. The further aim to reduce the negative effects of the host response to the bacilli on the blood supply to the brain is a further strong point of this study.

Because this is a protocol that has progressed very far into enrolment, not much can be changed in the manuscript or the reasoning, but it seems like a well thought through study. The reviewer has a few, mainly minor comments and suggested edits.

1. Abstract: To write out abbreviations at first use – "ATT"; "L(20)" – other drugs were abbreviated before, but not levofloxacin; "MRI"

2. Abstract: I am not convinced that the sub-studies "Nested …, and epilepsy" belong in the abstract, as these are just mentioned in a table, but no methods or detail are discussed in the main manuscript. Even if mentioned, the abbreviations "(PK)" and "(PG)" should be deleted (not used again)

3. Article summary; Strengths: Suggest add the word "randomised" to the first bullet point; last bullet under strengths – suggest write out "MRI"

4. Introduction, lines 8-9: The statement that in some settings up to 15% of children with TB have TBM is misleading: This was a study at two tertiary hospitals in a high TB-burden setting looking at children with culture-confirmed TB only and therefore is very biased towards more severely ill children with TB – I think the context need to be made clear.

5. Introduction, line 13: healthcare should be one word (or with hyphen) if followed by a noun

6. Introduction, last sentence page 4 over on to page 5: Were exposures of all of these drugs too low for TBM, or was isoniazid and pyrazinamide within acceptable range at higher end of suggested range?

7. High-dose rifampicin, Page 5: The child TBM study (reference 22) received only 30mg/kg/day for 8 weeks; the two adult studies used 35mg/kg/day - however, it is well known that children need higher mg/kg doses of RIF to achieve same concentrations/exposure than adults. Safety of higher RIF doses over longer durations therefore are not yet established in children – supposedly part of this study?

8. High-dose isoniazid, line 6: "metaboliser" should be "acetylator" (fast acetylator phenotype)

9. Levofloxacin as an alternativefourth agent,

- line 1: "plasma levels" should be "plasma concentrations" – for drugs in body fluids "concentrations" are preferred to "levels".

- In 3rd line "inhA" as a mutation should be in italics.

- In the last 3 lines, referring to the adverse effects of levofloxacin in the TB CHAMP trial, the correct interpretation is actually that there were few severe adverse effects (many more minor AEs)

10. Page 7, 2nd paragr, line 4: In other parts of the manuscript the authors use "phase-2" as the style, not "phase II" – suggest consistency.

11: Page 8, Main study objective a) incomplete end of sentence: "… children aged 29 days to < 18 years of age.

12. Box 1:

- Maybe motivate why gastro-intestinal bleeding is a specific AE to look out for (asprin)

- Arthralgia and arthritis are AEs specifically quite common on the fluoroquinolones in adolescents – I suggest mention these here as well (as was done in Box 2 – not sure why not included here)

- Drug resistance identified on genotypic/phenotypic drug susceptibility testing (add "testing" to complete sentence)

- Last line – change "HIV-infected" to "HIV-positive" or "children living with HIV"

13. Screening procedures:

- Line 5: Suggest "possible toxicities"

- Line 7: Suggest change "guardian/carer" to "parent/guardian" as in line 2.

- Line 11: Table 3a contains all procedures/data collected, but does not specifically collect what is said here (GCS, neurological fallout) but only stage of TBM – should staging not be included in a box for readers to understand how staging decision is made?

- Lines 12-13: It is important to take note hat there are huge differences between CSF collected from lumbar puncture (below the obstruction) and from ventricles (above the obstruction). A further consideration is whether doing microscopy for acid-fast bacilli is at all worthwhile if nucleic acid amplification tests and culture is much more accurate/sensitive?

- Lines 14-15: What do the authors mean by "Positive isolates" – cultured isolates of Mycobacterium tuberculosis? Drug susceptibility also possible on CSF with NAATs? Write out Mycobacterium tuberculosis at first use.

- Page 11, line 3: "Participants will have a…"?

14. Antituberculosis regimen:

- line 2: Pyrazinamide in the supplementary table is also indicated as a 150mg dispersible tablet?

15. HIV co-infection: Usually with TBM, antiretroviral therapy is not recommended to start before 4 weeks because of the risk of intracranial immune reconstitution inflammatory syndrome – was this changed for the study to start from 2 weeks?

16. Concomitant drugs (steroids): There is quite a variation in dose of the corticosteroids – could this not influence outcome of the study, especially "aspirin" arm? Is the dose of steroids going to be taken into account in analysis?

17. Pyridoxine supplementation (page 12). Children with TBM are usually expected to receive isoniazid at high-dose or at least higher end of the dose range – it may well have been advisable to give pyridoxine to all children with TBM?

18. Study follow-up, page 12:

- Line 3: Are caregivers receiving medicines for a two-month period without follow-up to see whether medication is given in the later part of treatment? This may be risk to the child – or is there a follow-up system for checking on medication during this time?

- Line 6: Should toxicities not be checked at every visit – they don't only present in the first 8 weeks?

19. Withdrawal of trial treatment: It is not clear what is meant by "Drug resistance (ATT specific)" – If initially resistant to any of study drugs, this should be exclusion criteria – does this "withdrawal" refer to later discovery of resistance and/or development of resistance to any study drug during treatment?

20. Page 15, 2nd to last line: CRF to be written in full before abbreviating. Also page 17, 1st line – IDMC and TSC to be written in full before abbreviating.

21. Discussion:

- Suggest add "randomised" in first line – it does make the study stronger than just the number

- Page 21: The protocol does not specify whether the manufacturers of the drugs are all the same in all of the sites/countries. Drug quality and bioavailability of medicines in fixed-dose combination tablets or even in different formulations may differ and could influence outcome. Are the pharmacokinetic studies evaluating this if different manufacturers/formulations?

- Page 21, 3rd last line: Suggest change "childhood presentations" to "children presenting"

22. References: Suggest carefully check references for correct style/abbreviations of journal names

23. Table 2:

- Suggest keep wording in text of MS same in table; change "parent/legal carer" to "Parent/legal guardian"

- Exclusion criteria: Why are only contacts of rifampicin-resistant source patients excluded and not also other drugs used in the regimen – because these DSTs are not done?

- Pregnancy – suppose only at enrolment only?

24. Figure 1. From the protocol and the statistical analysis plan, the reviewer had the impression that 400 participants are needed – why only 350 in the figure?

25. Table S4: 3rd block down under "Definition": Should it be "An adverse reaction"?

**Reviewer**	**2**
**Name**	**Mathias, Sitarah**
**Affiliation**	**Boston Children's Hospital**

**Date**	**15-Sep-2024**
**COI**	**Nil**

Well written, clear and concise. Few minor clarificaitons/comments:

1. Please specify the procedure of obtaining consent including the provision or written information in the local language, written consent forms and arrangements made for the use of specified in-person or virtual interpreters in the methods section (some references have been made in passing in the Discussion section).

2. Please explain the details of "partially blinding" and the reasons for the same in the methods section. Its unclear what information is blinded and to which party, and how to overcome potential lapses - for instance, patients or data collectors might guess which regimen is being adminsitered basedon number of drugs and the length of treatment. I'm sure this has been thought of - it just needs to be more clearly explained in the methods section.

**Reviewer**	**3**
**Name**	**Perera Valdivia, Doriam Alejandrino**
**Affiliation**	**Hospital Militar Escuela Dr Alejandro Dávila Bolaños, Neurosurgery**
**Date**	**16-Sep-2024**
**COI**	**Any competing interest to declare.**

It is a good and necessary piece of work, the results of which will help with this health problem.

I would recommend explaining the following better:

- How did you conceal the randomization? Supposedly there is a section where you explained it but you really did not explain that.

- Justify why part of the treatment is open label (ATT regimen)

VERSION 1 - AUTHOR RESPONSE

Reviewer: 1
Dr. Simon Schaaf, Desmond Tutu TB Centre, Stellenbosch University
Comments to the Author:
Review MS: SHORTENED INTENSIVE TREATMENT FOR CHILDREN WITH TUBERCULOUS MENINGITIS (SURE): a study protocol
The study is an important one to lead the way for shorter treatment for tuberculous meningitis in children with medicines that are readily available in low- and middle-income countries. The further aim to reduce the negative effects of the host response to

the bacilli on the blood supply to the brain is a further strong point of this study.

Because this is a protocol that has progressed very far into enrolment, not much can be changed in the manuscript or the reasoning, but it seems like a well thought through study. The reviewer has a few, mainly minor comments and suggested edits.

1. Abstract: To write out abbreviations at first use – "ATT"; "L(20)" – other drugs were abbreviated before, but not levofloxacin; "MRI".

Thank You. This has now been amended.

2. Abstract: I am not convinced that the sub-studies "Nested ..., and epilepsy" belong in the abstract, as these are just mentioned in a table, but no methods or detail are discussed in the main manuscript. Even if mentioned, the abbreviations "(PK)" and "(PG)" should be deleted (not used again)

Thank You. The abbreviations PK and PG have now been deleted. Authors have included this in the abstract to highlight the rare opportunity to address other important research questions in this large paediatric trial. If the 6-month ATT and high-dose randomisation are found to be efficacious, treatment would need to be provided alongside upscaling of diagnosis and evaluation of long term neurodevelopment. Other substudies including PK/PD and MRI substudy also provide important insights for drug dosing and improved understanding of pathophysiology which may direct future host directed therapy. A paragraph on nested substudies with reference to the table now occurs in the methods of the main text (previously in discussion). See pg 11 & pg 12 line 1-5.

3. Article summary; Strengths: Suggest add the word "randomised" to the first bullet point; last bullet under strengths – suggest write out "MRI"

Thank You. This has now been amended on pg 4.

4. Introduction, lines 8-9: The statement that in some settings up to 15% of children with TB have TBM is misleading: This was a study at two tertiary hospitals in a high TB-burden setting looking at children with culture-confirmed TB only and therefore is very biased towards more severely ill children with TB – I think the context need to be made clear.

Thank You for highlighting. This has now been amended. See pg 4, line 13-14.

5. Introduction, line 13: healthcare should be one word (or with hyphen) if followed by a noun.

Amended. Pg 4 line 17.

6. Introduction, last sentence page 4 over on to page 5: Were exposures of all of

these drugs too low for TBM, or was isoniazid and pyrazinamide within acceptable range at higher end of suggested range?

It is unclear which study the reviewer is referring to. If it can be clarified, we would be able to respond appropriately. If this is referring to Donald et al. study, (ref 14), then both INH and PZA have good CSF penetration whereas whereas RIF at adult doses rarely reaches CSF concentrations exceeding the MIC but does so more when doses up to 20mg/kg are used. If this is referring to Thee et al. study (ref 12) in children<2 yrs on the revised, 2010 WHO dosage recommendations, target plasma concentrations of first line ATT drugs, achieve target concentrations of R,H and PZA.

7. High-dose rifampicin, Page 5: The child TBM study (reference 22) received only 30mg/kg/day for 8 weeks; the two adult studies used 35mg/kg/day - however, it is well known that children need higher mg/kg doses of RIF to achieve same concentrations/exposure than adults. Safety of higher RIF doses over longer durations therefore are not yet established in children – supposedly part of this study?

Thank You. This has now been amended. See pg 5, line 37-39.

8. High-dose isoniazid, line 6: "metaboliser" should be "acetylator" (fast acetylator phenotype)

Amended. Pg 6 line 7.

9. Levofloxacin as an alternative fourth agent,
- line 1: "plasma levels" should be "plasma concentrations" – for drugs in body fluids "concentrations" are preferred to "levels".

Amended. Pg 6 line 11.

- In 3rd line "inhA" as a mutation should be in italics.

Amended. Pg 6 line 14.

- In the last 3 lines, referring to the adverse effects of levofloxacin in the TB CHAMP trial, the correct interpretation is actually that there were few severe adverse effects (many more minor AEs)

Thank You for clarifying. This has now been amended. Pg 6, line 20.

10. Page 7, 2nd paragr, line 4: In other parts of the manuscript the authors use "phase-2" as the style, not "phase II" – suggest consistency.

Thank You. Have reviewed and now use numerical style throughout. See pg 7, line 7.

11: Page 8, Main study objective a) incomplete end of sentence: "… children aged 29 days to < 18 years of age.

Amended. Pg 7, line 39.

12. Box 1:
- Maybe motivate why gastro-intestinal bleeding is a specific AE to look out for (asprin)
Because there is a potential increased risk of GI bleeding associated with prolonged intake of high dose aspirin, GI bleeding of any grade has been included as a specific AE in the trial. This has now been added to the manuscript. Pg. 8. Box 1.
- Arthralgia and arthritis are AEs specifically quite common on the fluoroquinolones in adolescents – I suggest mention these here as well (as was done in Box 2 – not sure why not included here).
Muscle/joint aching/pain i.e arthralgia and arthritis (grade 2 or above will be reported as events of interest as in Box 2. Those which are grade 3 or 4 will be reported as secondary safety outcomes (Box 1). This has now been added to Box 1 for clarity.

- Drug resistance identified on genotypic/phenotypic drug susceptibility testing (add "testing" to complete sentence).

Thank You. This has now been added.

- Last line – change "HIV-infected" to "HIV-positive" or "children living with HIV" .

This has now been amended.

13. Screening procedures:
- Line 5: Suggest "possible toxicities"

This has now been added. Pg 9 line 13

- Line 7: Suggest change "guardian/carer" to "parent/guardian" as in line 2.

Amended. Pg 9 line 15.

- Line 11: Table 3a contains all procedures/data collected, but does not specifically collect what is said here (GCS, neurological fallout) but only stage of TBM – should staging not be included in a box for readers to understand how staging decision is made?

Thank You, have now added as table footnote.

- **Lines 12-13: It is important to take note hat there are huge differences between CSF collected from lumbar puncture (below the obstruction) and from ventricles (above the obstruction). A further consideration is whether doing microscopy for**

acid-fast bacilli is at all worthwhile if nucleic acid amplification tests and culture is much more accurate/sensitive?

Agreed. Lumbar puncture is preferred, however in this pragmatic trial LP is not always practicable and ventricular CSF is taken instead taking into account it does not necessarily reflect lumbar CSF cell count and biochemistry. Also agree that microscopy for AFB has very low sensitivity however at some trial sites (HCMC in Viet Nam for example) AFB smear performs better (40-50% sensitivity) and the AFB smear can pick up cases which are missed by Xpert/culture. Where there is minimal CSF, Xpert/culture is prioritised. Most sites perform liquid culture. Given difficulty of confirming TBM and the absence of an adequately sensitive diagnostic test, AFB smear is included in this study as it also forms part of the definite TBM criteria as defined by the Uniform clinical case definition for research (Marais 2010).

- Lines 14-15: What do the authors mean by "Positive isolates" – cultured isolates of Mycobacterium tuberculosis? Drug susceptibility also possible on CSF with NAATs? Write out Mycobacterium tuberculosis at first use.
Positive isolates are cultured isolates of *M. tuberculosis* identified through either liquid or solid culture. Most sites have liquid culture and perform this routinely if there is enough CSF and/or supplies of MGIT bottles allow. Pg. 9 line 24.

In this trial Xpert MTB/Rif, Xpert Ultra or TrueNAT are the available NAAT and are performed directly on CSF. See Pg 9 line 23-25.

- Page 11, line 3: "Participants will have a…"? Amended. Pg 10, line 9

14. Antituberculosis regimen:
- line 2: Pyrazinamide in the supplementary table is also indicated as a 150mg dispersible tablet?

Yes this is correct.

15. HIV co-infection: Usually with TBM, antiretroviral therapy is not recommended to start before 4 weeks because of the risk of intracranial immune reconstitution inflammatory syndrome – was this changed for the study to start from 2 weeks.

Thank You. These protocol recommendations are taken from WHO 2016 guidelines at the time of writing however in keeping with revised WHO guidance, the protocol was updated to state that 'antiretroviral therapy should be delayed at least four weeks (and initiated within eight weeks) after treatment for TBM is initiated. This has been amended. Pg 11, line 14.

16. Concomitant drugs (steroids): There is quite a variation in dose of the corticosteroids – could this not influence outcome of the study, especially "aspirin" arm? Is the dose of steroids going to be taken into account in analysis?

Prednisone is included in both ATT treatment arms and we do not think that the variable dose of 2-4mg/kg daily is likely to impact on outcome. However, it will be reviewed in the final analysis to ensure that there are no significant differences in dose between arms and adjusted for in the analysis if necessary.

.

17. Pyridoxine supplementation (page 12). Children with TBM are usually expected to receive isoniazid at high-dose or at least higher end of the dose range – it may well have been advisable to give pyridoxine to all children with TBM?

As far as we authors are aware, INH induced peripheral neuropathy in children is rare and thus we have taken a pragmatic approach and followed WHO guidance on pyridoxine supplementation. This approach also ensures there is no bias with the shortened intensive ATT group receiving additional therapy that the standard 12-month group do not receive. Pyroxidine supplementation in children who are on a modified regimen consisting of additional cycloserine or linezolid is however recommended.

18. Study follow-up, page 12:
- Line 3: Are caregivers receiving medicines for a two-month period without follow-up to see whether medication is given in the later part of treatment? This may be risk to the child – or is there a follow-up system for checking on medication during this time?

In the later part of the trial, caregivers are receiving medicines for a two-month period without in-person follow-up in between. There are check-up phone calls made by research staff made regularly throughout the trial. Participants/carers have 24/7 access to research nurses should there be concern. This was designed in the study because less ATT or disease associated complications occur at this time and therefore less intensive monitoring required. Adherence and drug accountability is obtained at every follow-up visit.

- Line 6: Should toxicities not be checked at every visit – they don't only present in the first 8 weeks?

This has been clarified that toxicities are checked at every visit but blood tests to check for toxicities in the first 2 months and clinical monitoring throughout. Further testing is done if clinically indicated. See Pg 11, line 17-18.

19. Withdrawal of trial treatment: It is not clear what is meant by "Drug resistance (ATT specific)" – If initially resistant to any of study drugs, this should be exclusion criteria – does this "withdrawal" refer to later discovery of resistance and/or development of resistance to any study drug during treatment?

Drug resistance (ATT specific) refers to baseline resistance discovered after a participant has been randomised. These children would become late exclusions but will remain in the ITT analysis. No child who is randomised is excluded from analysis unless they are found to have an alternative diagnosis.

20. Page 15, 2nd to last line: CRF to be written in full before abbreviating. This has now been amended. Pg 13, line 30**. Also page 17, 1st line – IDMC and TSC to be written in full before abbreviating**. This has now been amended. Pg 18, line 14 and 16

21. Discussion:
- Suggest add "randomised" in first line – it does make the study stronger than just the number

This has now been added. Pg 15, line 26

- Page 21: The protocol does not specify whether the manufacturers of the drugs are all the same in all of the sites/countries. Drug quality and bioavailability of medicines in fixed-dose combination tablets or even in different formulations may differ and could influence outcome. Are the pharmacokinetic studies evaluating this if different manufacturers/formulations?

Manufacturers of the trial ATT are the same for all sites/countries. Pg 10. Line 19

- **Page 21, 3rd last line: Suggest change "childhood presentations" to "children presenting"**

See discussion section which has now been shortened and moved in the main text before ethics and dissemination as per editor's request. As this has been shortened, 'childhood presentations' has been deleted.

22. References: Suggest carefully check references for correct style/abbreviations of journal names.

This has been reviewed and amended to ensure consistency.

23. Table 2:
- Suggest keep wording in text of MS same in table; change "parent/legal carer" to "Parent/legal guardian"

Amended.

- Exclusion criteria: Why are only contacts of rifampicin-resistant source patients excluded and not also other drugs used in the regimen – because these DSTs are not done?

Correct, because the only molecular DST performed on CSF which is widely available in most LMICs is Xpert/MTB RIF and therefore only rifampicin resistance is determined at enrolment. If on phenotypic DST, resistance to other drugs is discovered, the child's study trestment is withdrawn and excluded from per protocol analysis but she/he stays in the trial for ITT analysis.

Any potential participant who has been in contact with person(s) who carry multi-drug resistance is likely to be excluded from recruitment folowing consultation with MRC CTU.

- Pregnancy – suppose only at enrolment only? Yes this is correct.

24. Figure 1. From the protocol and the statistical analysis plan, the reviewer had the impression that 400 participants are needed – why only 350 in the figure?

Amended with figure footnote.

25. Table S4: 3rd block down under "Definition": Should it be "An adverse reaction"? -This has now been amended

<u>Reviewer: 2</u>
Dr. Sitarah Mathias, Boston Children's Hospital
Comments to the Author:
Well written, clear and concise. Few minor clarifications/comments:

1. Please specify the procedure of obtaining consent including the provision or written information in the local language, written consent forms and arrangements made for the use of specified in-person or virtual interpreters in the methods section (some references have been made in passing in the Discussion section).

Thank You. This has now been further clarified and added. Consent is obtained in local language only. See Pg 16 lines 41-43

2. Please explain the details of "partially blinding" and the reasons for the same in the methods section. Its unclear what information is blinded and to which party, and how to overcome potential lapses - for instance, patients or data collectors might guess which regimen is being adminsitered basedon number of drugs and the length of treatment. I'm sure this has been thought of - it just needs to be more clearly explained in the methods section.

Further elaboration now provided on pg 7, lines 9-10.

<u>Reviewer: 3</u>
Dr. Doriam Alejandrino Perera Valdivia, Hospital Militar Escuela Dr Alejandro Dávila Bolaños
Comments to the Author:
It is a good and necessary piece of work, the results of which will help with this health problem.

I would recommend explaining the following better:
- How did you conceal the randomization? Supposedly there is a section where you explained it but you really did not explain that.

This is further clarified and details now added. See Pg 9, lines 39-40.

- Justify why part of the treatment is open label (ATT regimen)

Providing blinded ATT would require an enormous number of placebo formulations to be made which is not at all practical, particularly as dosages and formulations change with age. Furthermore it would defeat the purpose of providing benefit to families and patients if all children had to take either drug or placebo for a full year. Part of the benefit we hope to see is improved adherence in the 6 month arm because it is shorter.

VERSION 2 - REVIEW

**Reviewer**	**1**
**Name**	**Schaaf, Simon**
**Affiliation**	**Desmond Tutu TB Centre, Stellenbosch University, Paediatrics and Child Health**
**Date**	**22-Feb-2025**
**COI**	

Review: IS SHORTENED INTENSIVE TREATMENT FOR CHILDREN WITH TUBERCULOUS MENINGITIS EFFECTIVE AND SAFE (SURE)?: a protocol for a phase 3 randomised controlled trial evaluating 6 months of anti-tuberculosis therapyi and 8 weeks of aspirin in Asian and African children with tuberculous meningitis (Revised version)

The authors have satisfactorily addressed the reviewers' comments and suggested changes, and the reviewer thinks that the manuscript is acceptable as it is. However, a few very minor corrections may improve the manuscript – for the editor to decide (page numbers refer to clean version):

- Page 7, line 1: Suggest change to: "…essential to decrease the time to culture conversion."

- Page 7, line 14: "…exposures, safety of high rifampicin doses over longer durations is not yet established." (safety … is)

- Page 8, line 10: "High-dose aspirin…"

- Box 1, TB Treatment failure: "… TBM and/or TB disease at…" (move /);

and further down "Gastrointestinal bleeding…high-dose aspirin" (Hyphen as followed by a noun);

Last line in box: Suggest change "HIV-infected children" to "children living with HIV"

- Page 12, line 10: Mycobacterium tuberculosis (lowercase "t" for tuberculosis)

- Page 14, line 9: ART already abbreviated in line 7 above – use "ART"

- Page 15: Lines 26-27: should be no break in sentence – same line

- Page 17, line 23: I suppose this "Withdrawal of consent for (trial) treatment by patient" should include parents/guardians for younger children or children who cannot decide for themselves?

- Page 25, line 7: "facilities/clinics" (plural for clinics as well)

- Table 3: Abbreviations in table to be explained in footnote to the table

---

VERSION 2 - AUTHOR RESPONSE

Reviewer: 1
Dr. Simon Schaaf, Desmond Tutu TB Centre, Stellenbosch University
Comments to the Author:
Review: IS SHORTENED INTENSIVE TREATMENT FOR CHILDREN WITH TUBERCULOUS MENINGITIS EFFECTIVE AND SAFE (SURE)?: a protocol for a phase 3 randomised controlled trial evaluating 6 months of anti-tuberculosis therapy and 8 weeks of aspirin in Asian and African children with tuberculous meningitis (Revised version)

The authors have satisfactorily addressed the reviewers' comments and suggested changes, and the reviewer thinks that the manuscript is acceptable as it is. However, a few very minor corrections may improve the manuscript – for the editor to decide (page numbers refer to clean version):
Thank You for the comprehensive review and feedback. All suggested changes (as mentioned below) have now been made.
- Page 7, line 1: Suggest change to: "…essential to decrease the time to culture conversion."
Amended, page 5, line 31
- Page 7, line 14: "…exposures, safety of high rifampicin doses over longer durations is not yet established." (safety … is) Amended, page 6, line 1
- Page 8, line 10: "High-dose aspirin…" Amended, page 6, line 27
- Box 1, TB Treatment failure: "… TBM and/or TB disease at…" (move /); Amended.
and further down "Gastrointestinal bleeding…high-dose aspirin" (Hyphen as followed by a noun); Amended.
Last line in box: Suggest change "HIV-infected children" to "children living with HIV"
Amended.
- Page 12, line 10: Mycobacterium tuberculosis (lowercase "t" for tuberculosis) Amended.
Page 9, line 29
- Page 14, line 9: ART already abbreviated in line 7 above – use "ART" Amended. Page 11, line 2
- Page 15: Lines 26-27: should be no break in sentence – same line Amended.
- Page 17, line 23: I suppose this "Withdrawal of consent for (trial) treatment by patient" should include parents/guardians for younger children or children who cannot decide for themselves? Thank You, this is correct. This has now been amended to include parents/carers. Page 13, line 35
- Page 25, line 7: "facilities/clinics" (plural for clinics as well) Amended. Page 18, line 39
- Table 3: Abbreviations in table to be explained in footnote to the table. Thank You. Now included as footnote under table 3.
